# A small azide-modified thiazole-based reporter molecule for fluorescence and mass spectrometric detection

**DOI:** 10.3762/bjoc.10.258

**Published:** 2014-10-23

**Authors:** Stefanie Wolfram, Hendryk Würfel, Stefanie H Habenicht, Christine Lembke, Phillipp Richter, Eckhard Birckner, Rainer Beckert, Georg Pohnert

**Affiliations:** 1 Institute for Inorganic and Analytical Chemistry, Friedrich Schiller University, Lessingstr. 8, 07743 Jena, Germany; 2 Institute of Organic Chemistry and Macromolecular Chemistry, Friedrich Schiller University, Humboldtstr. 10, 07743 Jena, Germany; 3 Institute for Physical Chemistry, Friedrich Schiller University, Helmholtzweg 4, 07743 Jena, Germany

**Keywords:** activity-based protein profiling (ABPP), bioorthogonal, click chemistry, mass defect, molecular probe

## Abstract

Molecular probes are widely used tools in chemical biology that allow tracing of bioactive metabolites and selective labeling of proteins and other biomacromolecules. A common structural motif for such probes consists of a reporter that can be attached by copper(I)-catalyzed 1,2,3-triazole formation between terminal alkynes and azides to a reactive headgroup. Here we introduce the synthesis and application of the new thiazole-based, azide-tagged reporter 4-(3-azidopropoxy)-5-(4-bromophenyl)-2-(pyridin-2-yl)thiazole for fluorescence, UV and mass spectrometry (MS) detection. This small fluorescent reporter bears a bromine functionalization facilitating the automated data mining of electrospray ionization MS runs by monitoring for its characteristic isotope signature. We demonstrate the universal utility of the reporter for the detection of an alkyne-modified small molecule by LC–MS and for the visualization of a model protein by in-gel fluorescence. The novel probe advantageously compares with commercially available azide-modified fluorophores and a brominated one. The ease of synthesis, small size, stability, and the universal detection possibilities make it an ideal reporter for activity-based protein profiling and functional metabolic profiling.

## Introduction

Fluorescent dyes are widely used for detection and monitoring in the fields of chemistry, biochemistry, molecular biology, medicine and material sciences. Due to sensitive and selective detection methods and unproblematic toxicology they have almost completely replaced radioactive tags. Widely used representatives include dansyl chloride, fluoresceins, rhodamines and boron-dipyrromethenes (BODIPYs) [1]. Dansyl chloride, with a maximum UV–vis absorption at 369 nm, is one of the first extrinsic fluorescent dyes introduced in this field and is still widely used in protein labeling [2]. Later, fluoresceins and rhodamines found applications in this area as well because of advantageous UV–vis absorption maxima (480–600 nm) and more bathochromic emission wavelengths (510–615 nm) [3].

A successful class of fluorophores also used for probing in life science comprises the heterocyclic thiazoles. This structural element can be found in commercial products, such as thiazole orange, SYBR^®^ Green I or TOTO^®^, which are, e.g., used for DNA labeling. In these compounds the thiazole ring is part of a benzothiazole. We set out to minimize the structural complexity of the fluorophores to achieve higher atom economy and reduce the interaction with biomacromolecules. In this context it was critical to realize that the thiazole moiety itself can also act as a fluorophore, especially the class of 4-hydroxythiazoles [4–5]. 4-Hydroxythiazoles are now becoming commercially available but are also easily accessible by synthesis with a broad range of substitution patterns. Substantial manipulations of the UV–vis excitation and emission wavelengths of these compounds are thus possible [6].

The design of molecular probes based on fluorophores requires the attachment of the fluorescent reporter to bio(macro)molecules or synthetic probes. Especially “click chemistry”, introduced by Sharpless and coworkers in 2001 [7], is a widely used strategy to attach fluorophores covalently to other molecules. Among "click" reactions the Cu(I)-catalyzed azide–alkyne cycloaddition (CuAAC) is often considered as the prototypical transformation [7–9]. Due to the mild conditions and the use of aqueous solvents it is an efficient tool for bioorthogonal chemistry even inside of living systems [10]. One application of this concept for functional analysis of proteins is the activity-based protein profiling (ABPP) [11–12]. This proteomic strategy uses small probes designed to target active members of enzyme families [13]. These are often based on natural products to investigate their protein targets and eventually their mode of action [14–15]. ABPP probes contain two structural units: (1) a reactive group that reacts with the protein target and (2) a reporter unit for detection which could be, e.g., a fluorophore, a MS-tag, biotin or a combination of these [16–17]. For in vivo or in situ applications the alkyne (or azide) modified reactive group is usually applied to living organisms and after cell lysis the reporter is introduced by CuAAC [16]. Fluorophore tagged proteins can then be visualized by gel electrophoresis [17].

Besides fluorescence detection, mass spectrometry (MS) is also suited for the monitoring of tagged biological samples. Several probes have been designed for use with liquid chromatography–mass spectrometry (LC–MS). The probes attach covalently to target functional groups like amines, aldehydes/ketones, carboxylic acids and enhance their detection limit in LC–electrospray ionization (ESI) MS. This can be achieved by introduction of charged species like ammonium or phosphonium for ionization in the positive mode [18–20]. Bromine [19,21–22] or chlorine [23] containing tags were also introduced as they generate a unique isotopic pattern and therefore enhance recognition and identification of labeled small molecules. These specific isotopic patterns also enable data processing by cluster analysis [19] or other algorithms for an automated structure mining [24–25].

Here we introduce the rational design, synthesis and application of a small thiazole-based, azide-tagged reporter molecule that supports universally, fluorescence, UV and MS detection. We thoroughly characterize its reactivity and utility with different detection methods and compare it with common commercially available fluorophores. As proof of principle protein and amino acid labeling with an alkyne containing reactive probe according to the ABPP concept is introduced using a new reporter molecule.

## Results and Discussion

### Design of the reporter

**Figure 1:**
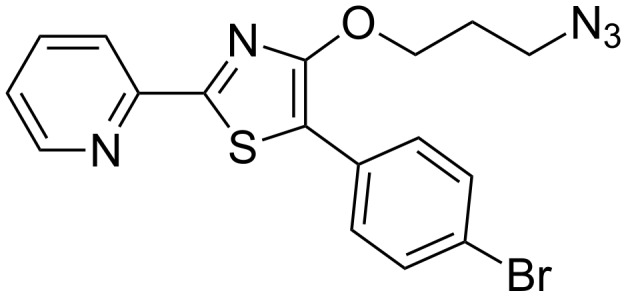
Structure of the reporter molecule BPT (**1**).

We aimed to combine high UV absorption and fluorescence with the possibility of unambiguous mass spectrometric detection (LC–ESIMS) in one reporter molecule. An azide functionality guarantees compatibility with widely applicable CuAAC approaches, that are, for instance, used in the field of ABPP where fluorescent reporter azides act as part of protein probes. To avoid the need for expensive detection systems for in-gel fluorescence we adjusted the excitation and emission wavelengths of the reporter to basic laboratory documentation equipment (365 nm UV-transilluminator, digital camera and a low cost commercial UV filter). Introduction of at least one atom with characteristic isotopic pattern like bromine or chlorine is necessary for a unique mass spectrometric detection of labeled substances. However, introduction of these heavy atom substituents in a fluorophore is challenging since it often results in decreased fluorescence due to intersystem crossing [26]. When working with reversed-phase LC–MS not only a specific isotopic pattern but also balanced polarity of the reporter is required. Addition of a nonpolar reporter shifts the retention time of polar analytes in reversed-phase chromatography to higher values. This is especially advantageous in the detection of small polar analytes [23,27]. On the other hand the polarity of the reporter needs to be high enough to work with biological samples in aqueous solution. Ideally a pH-independent fluorescence should guarantee for unbiased detection in tissues or under variable analytical conditions. In the light of our detailed knowledge of luminescence properties of pyridylthiazoles we considered this compound class to be ideally suited for the above mentioned tasks [28]. Based on previous considerations on the luminescent properties of pyridylthiazoles we decided to synthesize 4-(3-azidopropoxy)-5-(4-bromophenyl)-2-(pyridin-2-yl)thiazole (BPT, **1**, Figure 1) as target molecule fulfilling the above mentioned requirements.

### Synthesis

**Scheme 1:**
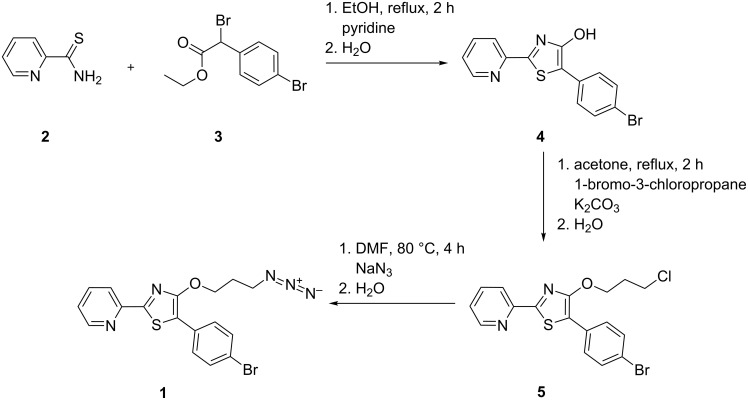
Synthesis of the azide-bearing 4-hydroxythiazole derivative **1**.

The synthesis of the azide-bearing fluorophore starts with a Hantzsch thiazole formation employing pyridine-2-carbothioamide (**2**) and ethyl 2-bromo-2-(4-bromophenyl)acetate (**3**) (Scheme 1). The cyclization reaction leads, under basic catalysis in moderate yield (ca. 50%), to 5-(4-bromophenyl)-2-(pyridin-2-yl)thiazol-4-ol (**4**). This derivative of the 4-hydroxythiazole family was already synthesized by Beckert et al. in a study focusing on the fluorescence properties of 4-hydroxythiazoles [29]. It exhibits an intense bathochromic shift of the UV–vis absorption when deprotonated at the 4-hydroxy position. The reactive 4-hydroxy position is alkylated employing 1-bromo-3-chloropropane in acetone, yielding the chloropropyl ether **5** in a good yield (85%). The chlorine in compound **5** is subsequently exchanged using an excess of sodium azide in DMF at 80 °C for several hours, leading to the organic azide **1** in good yield (83%).

The thioamide **2** bears an electron-withdrawing substituent in form of a 2-pyridyl moiety, which is important for an efficient fluorescence of the final product [6]. The α-bromoester **3** bears a bromine atom at the 4-position of the phenyl ring, which is introduced to facilitate MS detection.

**Figure 2:**
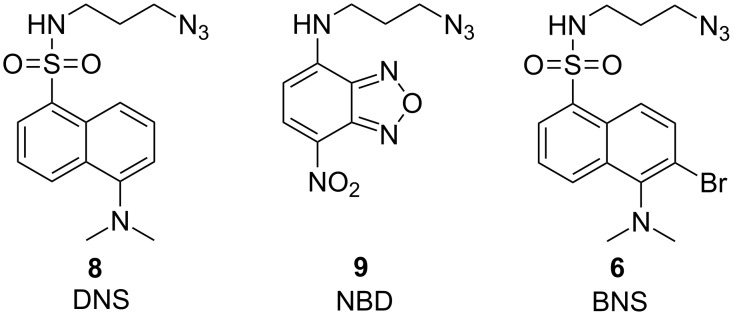
Structure of the tested azide-modified standard fluorophores DNS (**8**) and NBD (**9**) and the bromine modified DNS system **6**.

For comparison, we also synthesized and tested a bromine modified dansyl derivative *N*-(3-azidopropyl)-6-bromo-5-(dimethylamino)naphthalene-1-sulfonamide (BNS, **6**, Figure 2). Dansyl chloride is brominated according to the literature [30] to produce 6-bromo-5-(dimethylamino)naphthalene-1-sulfonyl chloride (**7**) and subsequently treated with 3-azidopropan-1-amine to provide the fluorescence/MS tag **6**.

### Characterization of BPT (**1**) and comparison with other azide modified fluorophores

We characterized the new thiazole reporter BPT (**1**) regarding its absorption and emission properties as well as its quantum yield and compared it with other commercially available fluorophores of similar size (Figure 2). We chose *N*-(3-azidopropyl)-5-(dimethylamino)naphthalene-1-sulfonamide (DNS, **8**) with a fluorophore system exhibiting a large stokes shift [1] suitable for fluorescence detection with UV filters. Derivatization with dansyl chloride is used for labeling of primary and secondary amines or phenols resulting in enhanced ESI signals and shifted retention times of labeled polar analytes in reversed-phase LC [27]. For comparison with BPT (**1**) we also introduced bromine into the aromatic system of DNS (**8**) to receive BNS (**6**). Furthermore, we utilized *N*-(3-azidopropyl)-7-nitrobenzo[*c*][1,2,5]oxadiazol-4-amine (NBD, **9**), a cheap fluorophore previously used for probes [1,31] or as fluorescent tag [32–33] (Figure 2).

**Figure 3:**
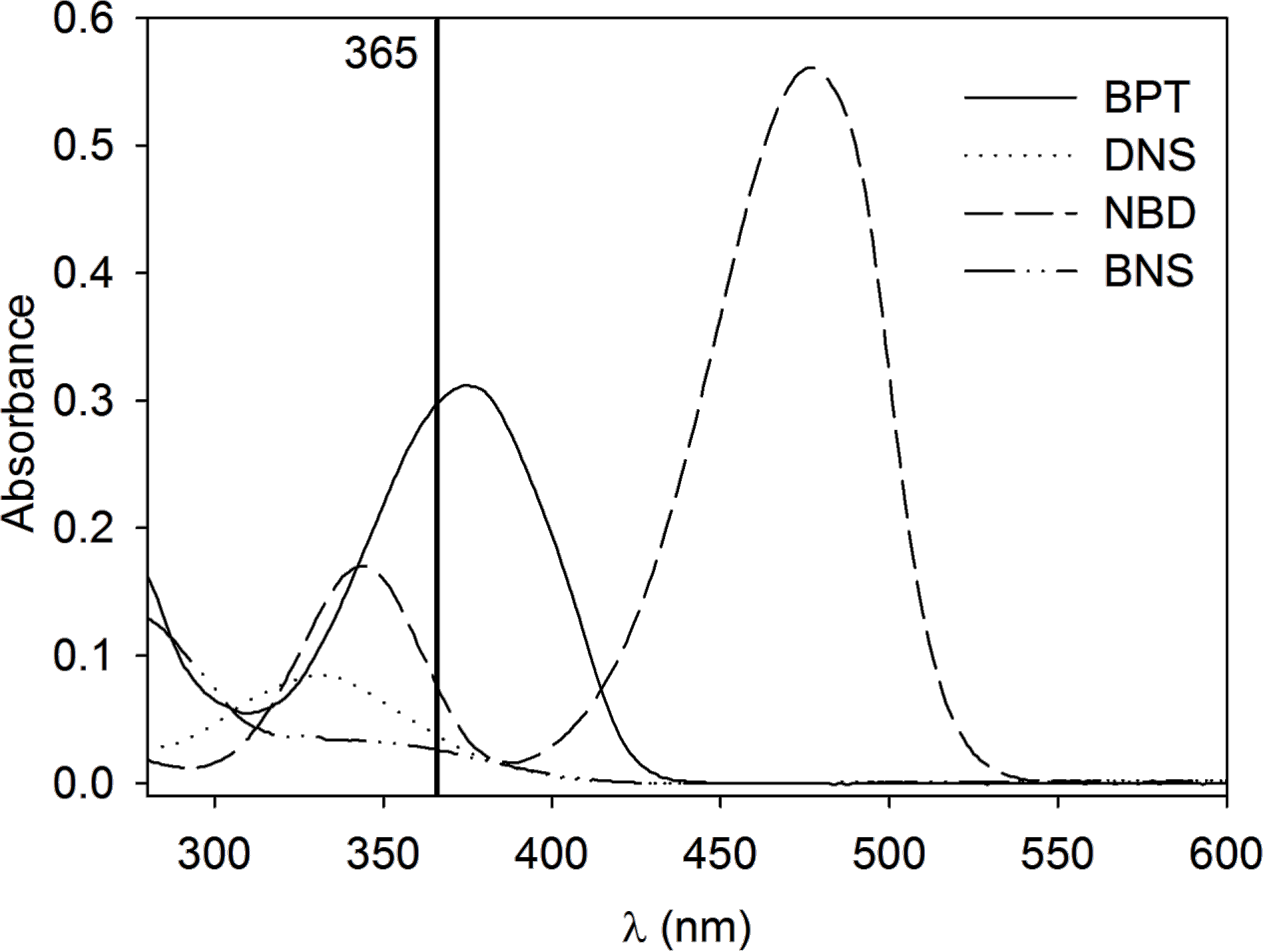
UV–vis spectra of 20 µM solutions of the azide modified fluorophores BPT (**1**), DNS (**8**), NBD (**9**) and BNS (**6**) in THF/water (20:80; v/v).

UV–vis spectra of all substances were recorded in an aqueous solution containing 20% THF (v/v) (Figure 3) and their molar absorption coefficients ε at their absorption maxima (λ_abs,max_) were calculated (Table 1). Notably, introduction of bromine into DNS (**8**) decreases the molar absorption coefficient more than twice and makes the resulting compound BNS (**6**) unsuitable for excitation with an UV transilluminator whereas BPT (**1**) offers a very good absorbance at 365 nm, a standard UV excitation wavelength.

**Table 1:** Spectral properties of BPT (**1**), DNS (**8**), NBD (**9**) and BNS (**6**) in THF/water (20:80; v/v).

	BPT (**1**)	DNS (**8**)	NBD (**9**)	BNS (**6**)

λ_abs,max_ [nm]	374 (376^a^)	331	344/477	327
ε [L/(mol cm)]	15.5 × 10^3^	4.3 × 10^3^	8.5 × 10^3 b^/28.1 × 10^3^	1.9 × 10^3^
λ_em,max_ [nm]	455 (444^a^)	546	545	550
Φ^c^	0.87 (0.96^a^)	n.d.	0.51^d^	<0.03

Lit. ε [L/(mol cm)]		4.2 × 10^3 e,f^	22.1 × 10^3 d^	
Lit. λ_em,max_ [nm]		506 nm^e,g^, 520 nm^e,f^	524 nm^d^	

^a^In cyclohexane; ^b^at 344 nm (which is a local maximum of NBD), since we excite fluorescently labeled biomolecules in gels with a 365 nm UV transilluminator this region is of crucial importance; ^c^quantum yield, ^d^in ethanol [45]; ^e^solvent not specified; ^f^from [46]; ^g^from [47]; n.d. - not determined; Lit. - values found in literature.

**Figure 4:**
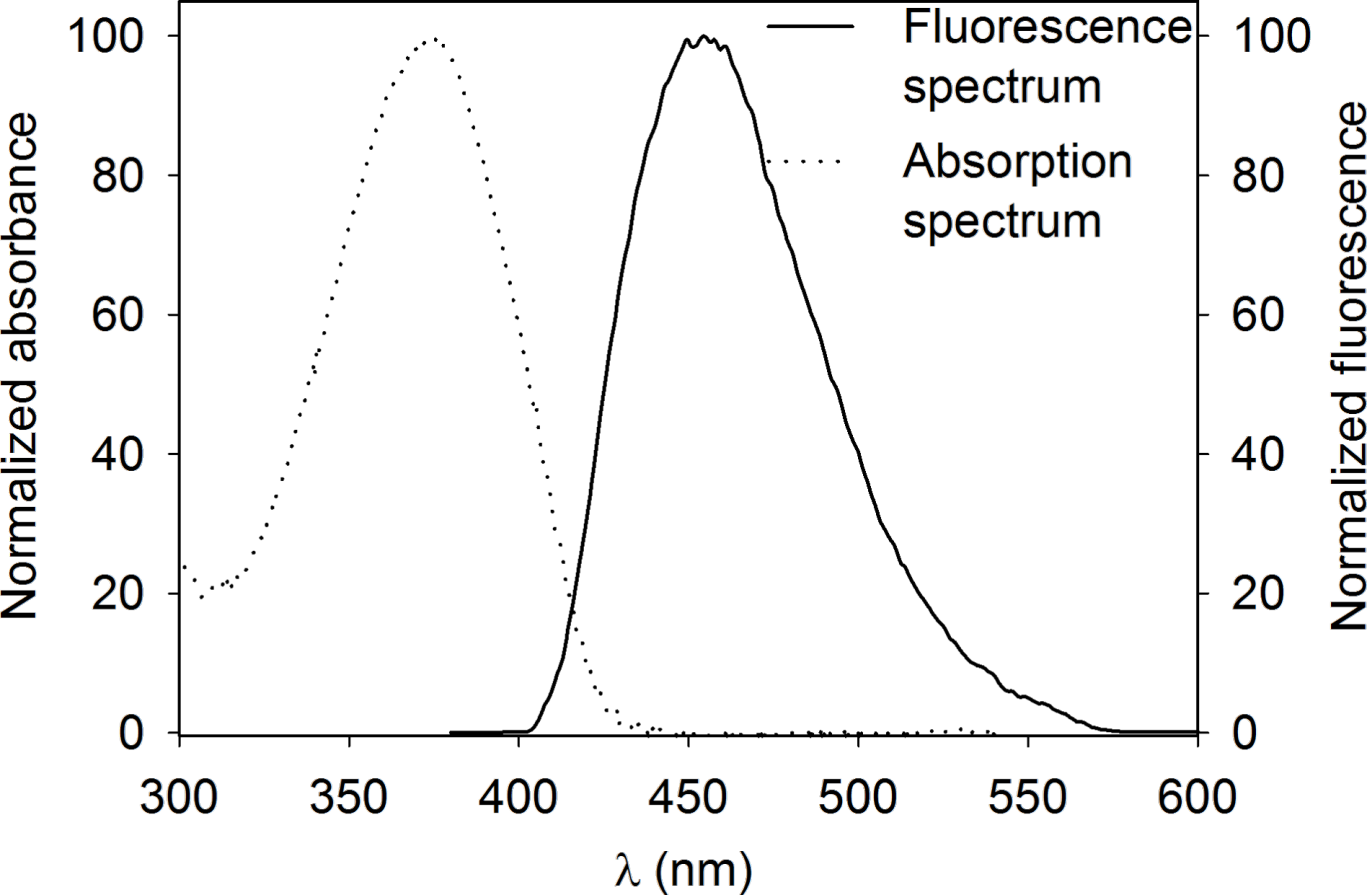
Normalized absorbance and fluorescence of BPT (**1**) in 20% THF/80% water (v/v), excitation at 374 nm.

We then recorded fluorescence spectra of all fluorophores and determined fluorescence quantum yields Φ (Table 1). To characterize BPT (**1**) in a non-interacting solvent we used cyclohexane resulting in an emission maximum of 444 nm and a quantum yield of Φ = 0.96. In an aqueous solution containing 20% THF (v/v) the maximum of emission (λ_em,max_) is shifted to 455 nm (Figure 4). The quantum yield of BPT (**1**) in this solution is Φ = 0.87 which makes it convenient for fluorescence detection in aqueous media, e.g., for biological applications. In contrast, the quantum yield of BNS (**6**) in 80% water/20% THF (v/v) was below 0.03 and rendering it inappropriate for this purpose.

**Figure 5:**
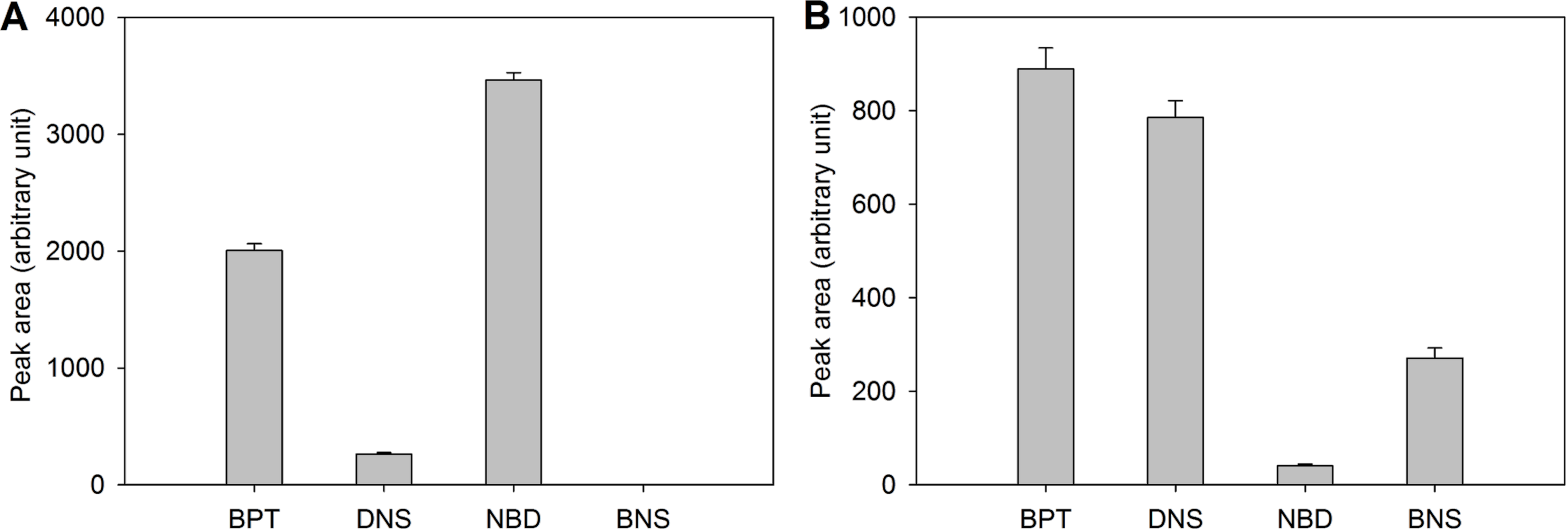
Peak area of 100 pmol BPT (**1**), DNS (**8**), NBD (**9**) and BNS (**6**) measured with (A) C18-UPLC coupled to a photodiode array detector at their absorption maxima or (B) C18-UPLC–ESIMS in positive ionization mode.

The UV properties allow UV detection after LC separation as could be shown by ultra-performance liquid chromatography (UPLC) coupled to a photodiode array detector using the solvents A (water/acetonitrile/formic acid 98:2:0.1; v/v/v) and B (acetonitrile/0.1% formic acid; v/v). The peaks of equimolar amounts were integrated at their absorption maxima resulting in the highest integrated peak area for NBD (**9**) followed by BPT (**1**) (Figure 5A). BPT is the least polar substance among the tested fluorophores and elutes at 90% B (Table 2). Polar analytes are often poorly retained in reversed-phase chromatography [23]. Thus, after CuAAC BPT (**1**) will shift retention times of polar analytes to higher values as it is achieved with other labeling reagents like dansyl chloride [27] or *p*-chlorophenylalanine containing tags [23]. Nevertheless, BPT (**1**) shows sufficient water solubility when working in aqueous solutions with low amounts of organic co-solvent. For instance during implementation of CuAAC we use 3.5% DMSO and 4.5% *t*-BuOH in our protocol which ensures solubility of BPT (**1**).

**Table 2:** Solvent composition at time of elution of BPT (**1**), DNS (**8**), NBD (**9**) and BNS (**6**) measured with C18-UPLC–ESIMS using a linear gradient of solvents A (water/acetonitrile/formic acid 98:2:0.1; v/v/v) and B (acetonitrile/0.1% formic acid; v/v) and solvent composition and masses of imines **11**–**14** formed in a model reaction between L-lysine and DDY (**10**) followed by CuAAC with the different reporter molecules.

reporter	comp. B (%)	imine	comp. B (%)	*m*/*z^a^*

BPT (**1**)	90	**11**	33	691.19
DNS (**8**)	58	**12**	20	609.31
NBD (**9**)	49	**13**	24	539.26
BNS (**6**)	74	**14**	31	687.22

^a^Calculated monoisotopic masses; comp. - solvent composition.

UPLC–ESIMS measurements were employed to characterize ionization properties of the fluorophores (Figure 5B). In positive mode BPT (**1**) gives a slightly higher peak area than DNS (**8**). Derivatization with dansyl chloride has been previously introduced as an ionization enhancing procedure for LC–ESIMS. Thereby increased linear responses of tested amino acids by over two orders of magnitude compared to underivatized samples were observed [27]. Interestingly, the novel BPT (**1**) is even superior to the established DNS (**8**) but introduced the additional benefit of a characteristic isotope pattern. Ionization of the brominated dansyl system BNS (**6**) resulted in a clearly lower intensity response. In negative mode ionization of BPT (**1**) is not adequate (data not shown).

Taken together in the comparison of all four fluorophores BPT (**1**) has superior properties for detection if fluorescence, UV absorption and MS properties are concerned.

### Visualization of small molecules by mass spectrometric detection

**Figure 6:**
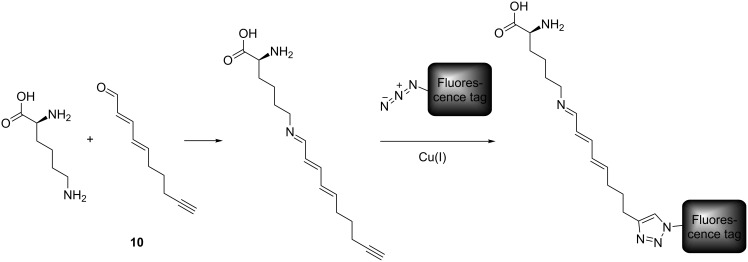
Procedure of the model reaction between L-lysine and DDY (**10**) to form an imine (only one of two possible reactions shown) followed by CuAAC with the azide-modified fluorophores. The rectangle represents the respective reporter unit. For clarity only reactions with the terminal lysine are depicted.

**Figure 7:**
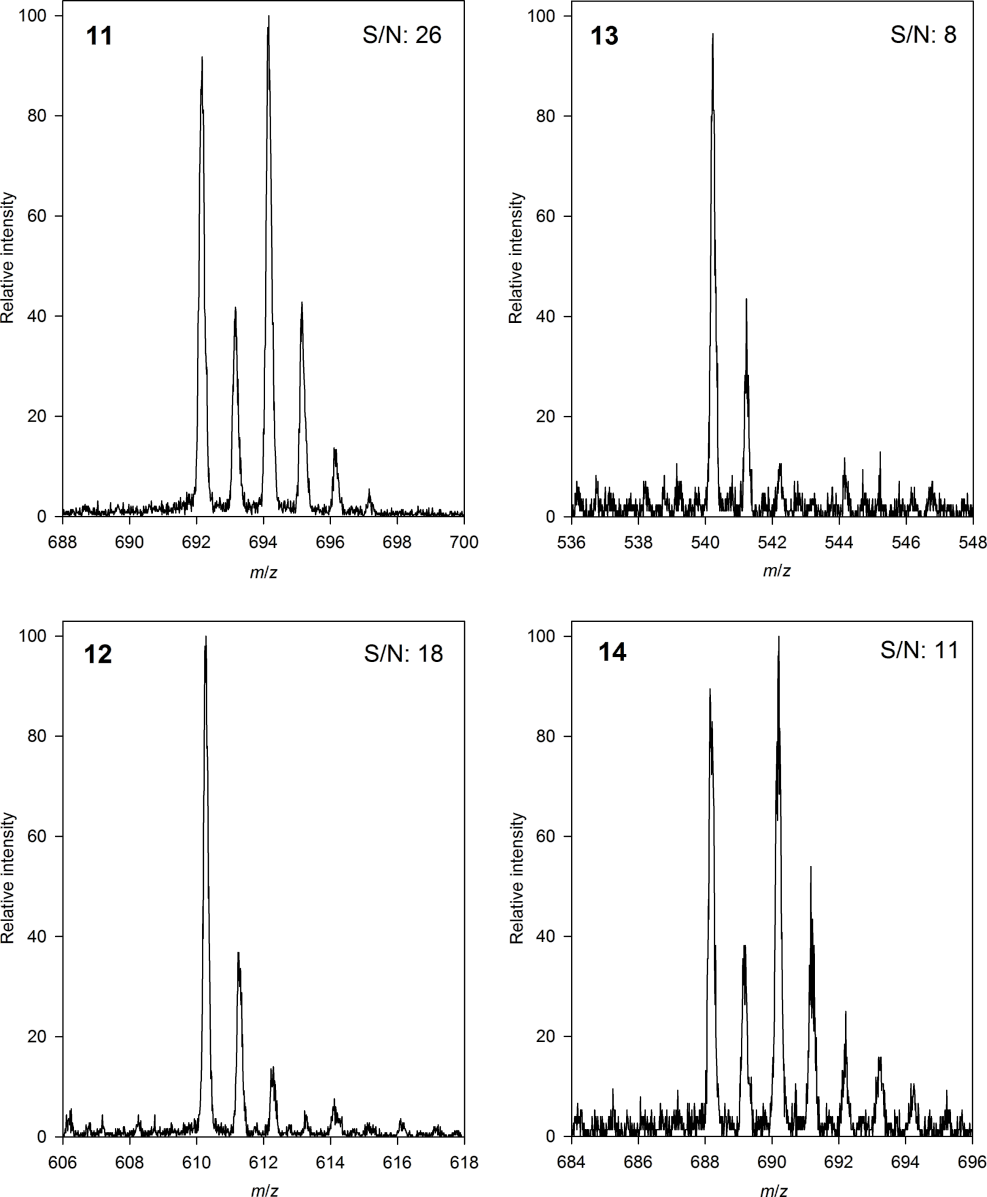
Mass spectra of labeled L-lysine/DDY (**10**)/fluorophore conjugates **11** (containing BPT), **12** (containing DNS), **13** (containing NBD) and **14** (containing BNS) measured in ESI positive mode (S/N: signal to noise ratio after reaction of equal molar amounts of reagents).

To demonstrate the universal application possibilities, we next coupled reporter molecules with a synthetic reactive group as commonly used in ABPP approaches. The alkyne-modified (2*E*,4*E*)-deca-2,4-dien-9-ynal (DDY, **10**) served as reactive group. DDY (**10**) mimics the natural product 2,4-decadienal that is produced by some diatoms as potential chemical defense metabolite against their grazers [34]. Structure-activity tests have revealed that 2,4-decadienal can be modified in the alkyl terminus without loss of function [35]. Thus the alkyne modified α,β,γ,δ-unsaturated aldehyde **10** can serve as a tool for the elucidation of the mode of action of the compound class of polyunsaturated aldehydes. DDY (**10**) was initially transformed with L-lysine to form an imine before CuAAC was performed with the four azides BPT (**1**), DNS (**8**), NBD (**9**) and BNS (**6**). After one hour of incubation with lysine the respective reporter, the ligand 1-(1-benzyltriazol-4-yl)-*N*,*N*-bis[(1-benzyltriazol-4-yl)methyl]methanamine (TBTA), sodium ascorbate and a copper sulfate solution were added and incubated for another hour (Figure 6). All four reactions were performed with identical molar amounts of the reagents. After centrifugation products were characterized with LC–ESIMS in positive mode (Table 2, Figure 7).

Mass spectra in Figure 7 show clearly that most intensive signals can be obtained with the novel reporter BPT (**1**). BPT (**1**) is thus transformed efficiently in the CuAAC reaction and the products such as **11** can be detected with high sensitivity using LC–MS.

Besides the coupling product of BPT (**11**) only the low fluorescent **14** shows unique isotopic patterns caused by the two isotopes ^79^Br and ^81^Br. This enables identification of tagged analytes even in complex samples. Introduction of bromine substituents does not only affect the isotopic pattern of analytes but also increases ionization and the detection limit of small metabolites [36] and peptides [37]. In addition, introduction of Br or Cl by labeling allows the application of cluster analysis [19] or other software [38] as computational tools to identify probe-reactive analytes out of complex mixtures even of unknown mass.

### Visualization of proteins by in-gel fluorescence detection

In a model reaction we tested the suitability of BPT (**1**) and the other reporters for in-gel fluorescence detection of labeled proteins. Since DDY is universally reactive against proteins, we chose arbitrary a catalase from bovine liver as target protein that was reacted with DDY (**10**).

**Figure 8:**
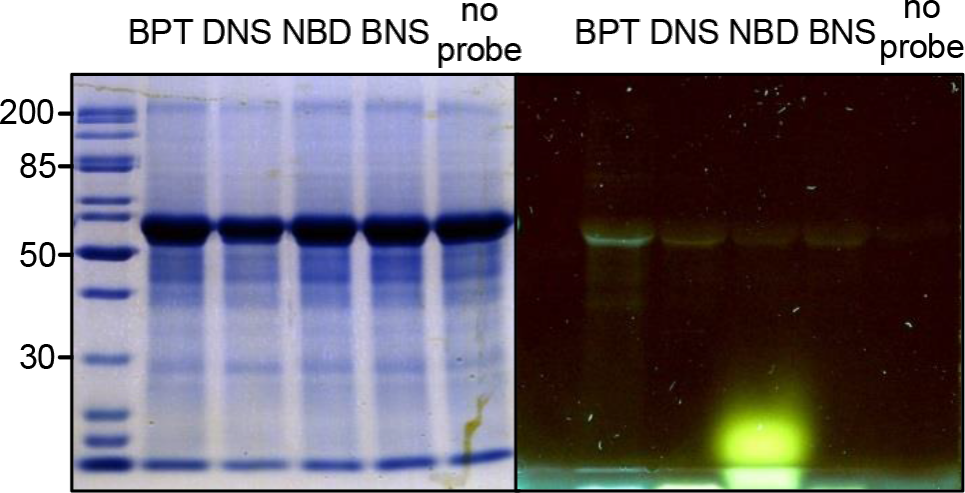
Fluorescent labeling of catalase treated with DDY (**10**) followed by CuAAC with all four reporter molecules and in-gel fluorescence detection at 365 nm. Equivalent amounts of protein were labeled and loaded on the gel. (Protein ladder masses in kDa).

After addition and incubation of DDY (**10**) with the catalase, we applied CuAAC with the four fluorophores. The products were then separated by sodium dodecyl sulfate polyacrylamide gel electrophoresis (SDS-PAGE) and visualized by in-gel fluorescence detection (Figure 8). BPT/DDY gives the brightest signal whereas intensities of DNS/DDY and BNS/DDY-labeled catalase are clearly lower. The lowest signal was emitted by NBD/DDY/catalase, which is probably due to very low fluorescence quantum yields reported for NBD derivatives of primary amines in water [39]. Furthermore, NBD (**9**) is not suitable for standard SDS-PAGE (12% gels) as the dye smears and therefore potentially covers fluorescent signals of proteins of lower masses.

The novel probe has further implications since a combination of fluorescence and mass tagging might prove beneficial in proteomics studies. Mass tags containing bromine [24,37,40–41] and chlorine [24,38] have been reported in proteomics related applications. Additionally, bromine containing tags called isotope-differentiated binding energy shift tags (IDBEST™) were designed to introduce a mass-defect in peptides for better sequence coverage of proteins [25,42]. The reporter molecule BPT (**1**) contains bromine and tagged proteins can thus easily be identified with both, fluorescent and MS techniques.

## Conclusion

We introduce the azide-modified thiazole-based reporter molecule BPT (**1**) with superior properties for fluorescence, UV and MS detection compared to other common reporters. BPT (**1**) can be easily synthesized and attached to terminal alkyne-modified molecules via CuAAC. We show model experiments that demonstrate the suitability of the molecule in labeling small molecules and in ABPP investigations. Fluorescence and MS offer orthogonal opportunities for detection and make this reporter a universal tool for targeting molecules of different sizes and properties.

## Experimental

### Synthesis

Experimental details are available in Supporting Information File 1.

### Sample preparation and measurements

#### UV–vis and fluorescence spectroscopy

Solutions of each fluorophore in THF/water (20 µM, 20:80; v/v) were prepared out of 5 mM stock solutions in DMSO. UV–vis spectra were recorded with a GENESYS™ 10 S spectrophotometer (Thermo Fischer Scientific Inc., Waltham, MA, USA) with 10 mm quartz cells. Quantum yields were obtained as described in [43] using quinine sulfate in 0.05 M sulfuric acid as fluorescent standard with a Varian Cary 500 spectrophotometer (Varian Inc., Palo Alto, CA, USA) in combination with a Luminescence Spectrophotometer LS 50 (Perkin Elmer, Waltham, MA, USA). For DNS (**8**) and NBD (**9**) emission maxima were obtained with a FP-6500 (Jasco, Tokyo, Japan) spectrofluorimeter.

#### LC–ESIMS and UV–vis detection

For LC–MS measurements we used an Acquity™ Ultraperformance LC (Waters, Milford, MA, USA) coupled to a Waters 996 PDA detector and a Q-Tof microMS (Waters Micromass, Manchester, England). A Kinetex C18 reversed-phase column (2.1 mm × 50 mm, 1.7 µm particle size, Phenomenex, Torrance, CA, USA) was used. For UV detection and ionization in positive and negative mode 10 µL of 10 µM solutions of each fluorophore in water containing 5% DMSO were injected. For model reactions between DDY (**10**) and lysine followed by CuAAC, 5 µL were injected.

#### Incubation with DDY (**10**) and CuAAC

**For L-lysine:** L-Lysine (30 µL 1 mM, prepared from a 50 mM stock in water) were added to 1.47 mL methanol followed by 4 µL (0.13 mM) of DDY (**10**) (prepared from a 50 mM stock in DMSO) and mixed on a vortex mixer. 132 µL of this solution were transferred to a 1.5 mL Eppendorf tube (Eppendorf AG, Hamburg, Germany) and the following substances were added (procedure adapted from [16]): 3 µL (0.1 mM) of BPT (**1**) or the other reporter molecules (5 mM stock in DMSO), 9 µL (0.1 mM) TBTA solution (1.7 mM stock in DMSO/*tert*-butanol, 1:4, v/v) and 3 µL (20 mM) freshly prepared ascorbic acid solution (1.00 M in water). Samples were vortexed and 1 µL (1 mM) copper(II) sulfate solution (from a 50 mM stock solution in water) was added. Samples were vortexed again, centrifuged after one hour and measured by UPLC–MS.

**For catalase from bovine liver:** Catalase from bovine liver (2.5 mg) was dissolved in 1 mL phosphate buffer (59.0 mM Na_2_HPO_4_, 7.6 mM KH_2_PO_4_, pH 7.6) and 2 µL (0.01 mM) of DDY (**10**, 5 mM stock in DMSO) were added. The sample was incubated for one hour. 44 µL of this solution were transferred to an Eppendorf tube and the following substances were added (procedure adapted from [16]): 1 µL (0.1 mM) of BPT (**1**) or the other reporter molecules (5 mM stock in DMSO), 3 µL (0.1 mM) TBTA solution (1.7 mM stock in DMSO/*tert*-butanol, 1:4, v/v) and 1 µL (20 mM) of a freshly prepared ascorbic acid solution (1.00 M in water). Samples were vortexed and 1 µL (1 mM) copper(II) sulfate solution (50 mM in water) was added. Samples were vortexed again and stored on ice for 1 hour.

#### SDS-PAGE and in-gel fluorescence detection

Aliquots (10 µL) of each pre-incubated catalase (9 µL of untreated catalase and 1 µL of deionized water) were mixed with 10 µL of 2× loading buffer [44] and heated to 95 °C for 6 min. A protein ladder (PageRuler unstained protein ladder, Thermo Scientific) and 15 µL of each sample were loaded on a 12% SDS mini gel and separated in a Mini-Protean^®^ Tetra gel cell (Bio-Rad, Herculas, CA, USA) by applying 80 V for 30 min followed by 180 V for 65 min. A fluorescent picture was taken at 365 nm irradiation using a UV transilluminator (Bio-Rad, UV star), a PowerShot A640 camera (Canon, Tokyo, Japan) and a commercially available UV filter (HMC Hoya Multi-Coated Filter, Hoya, Tokyo, Japan). The gel was stained with RAPIDstain™ (G-Biosciences, St. Louis, MO, USA).

## Supporting Information

File 1 Synthetic procedures and characterization data of synthetic compounds.
