# Supplementary material for: A small azide-modified thiazole-based reporter molecule for fluorescence and mass spectrometric detection
Source: Beilstein J Org Chem. 2014 Oct 23;10:2470–9. doi: 10.3762/bjoc.10.258 (PMC4222447; doi:10.3762/bjoc.10.258)
Supplement: File 1 — Synthetic procedures and characterization data of synthetic compounds. [file Beilstein_J_Org_Chem-10-2470-s001.pdf]

Supporting Information

for

A small azide-modified thiazole-based reporter molecule for fluorescence and mass spectrometric detection

Stefanie Wolfram[1], Hendryk Würfel[2], Stefanie H. Habenicht[2], Christine Lembke[1], Phillipp Richter[1], Eckhard Birckner[3], Rainer Beckert[2] and Georg Pohnert*[1]

Address: [1]Institute for Inorganic and Analytical Chemistry, Friedrich Schiller University, Lessingstr. 8, 07743 Jena, Germany and [2]Institute of Organic Chemistry and Macromolecular Chemistry, Friedrich Schiller University, Humboldtstr. 10, 07743 Jena, Germany and [3]Institute for Physical Chemistry, Friedrich Schiller University, Helmholtzweg 4, 07743 Jena, Germany

Email: Georg Pohnert* - Georg.Pohnert@uni-jena.de

* Corresponding author

Synthetic procedures and characterization data of synthetic compounds

Synthetic procedures

General methods

All chemicals were purchased as reagent grade or better and used without further purification. If necessary reactions were performed under argon. Commercially available dry solvents were employed. Diethyl ether and tetrahydrofuran (THF) contained butylhydroxytoluene as peroxidation inhibitor. Column chromatography was carried out on Merck silica gel (0.04–0.063 mesh). TLC was performed with TLC silica gel 60 F_{254} plates from Merck. TLC spots were visualized by irradiation of the TLC plate with UV radiation (254 nm) or by dipping in Seebach reagent (2.5 g phosphomolybdic acid and 1 g cer(IV) sulfate dissolved in 65 mL water and slowly acidified by dropwise addition of 6 ml concentrated sulfuric acid).

5-(4-Bromophenyl)-2-(pyridin-2-yl)thiazol-4-ol (4)

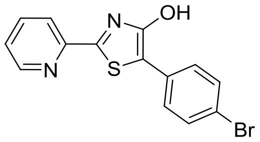

Starting from pyridine-2-carbothioamide (**2**) and ethyl 2-bromo-2-(4-bromophenyl) acetate (**3**) the compound was synthesized according to literature [1].

^{1}H NMR (400 MHz, DMSO-d_6): δ (ppm) 11.64 (s, 1H, OH), 8.62 (d, 1H, ArH), 8.03-7.90 (m, 2H, ArH), 7.72 (d, 2H, ^{3}J = 8.6 Hz, ArH), 7.58 (d, 2H, ^{3}J = 8.6 Hz, ArH), 7.50-7.45 (m, 1H, ArH).

^{13}C NMR (100 MHz, DMSO-d_6): δ (ppm) 160.7, 158.9, 149.8, 149.5, 137.4, 131.4, 131.0, 127.7, 124.7, 118.8, 118.2, 109.0.

MS (EI): *m/z* 333.9 [42%] M+2, 331.9 [42%] M, 263.9 [29%], 218.9 [100%], 200.7 [58%].

HRMS: *m/z* calculated: 331.9619, found: 331.9617.

5-(4-Bromophenyl)-4-(3-chloropropoxy)-2-(pyridine-2-yl)thiazole (5)

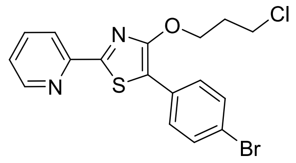

In a 100 mL Erlenmeyer flask 0.81 g (2.4 mmol) **4**, 0.5 g (3.6 mmol) K_2CO_3 and 0.3 mL (3.0 mmol) 1-bromo-3-chloropropane were stirred in 20 mL DMF at r.t. for 6 h. The mixture was poured in 200 mL of water and extracted with $CHCl_3$ (3 x 50 mL). The extracts were combined, washed with saturated K_2CO_3 solution and water, dried over $MgSO_4$ and evaporated in vacuum to obtain a bright yellow solid. The compound was recrystallized from heptane/$CHCl_3$ to obtain light yellow crystalls in 85% yield.

^{1}H NMR (250 MHz, $CDCl_3$): δ (ppm) 8.59 (d, 1H, ^4J = 4.6 Hz, ArH), 8.11 (d, 1H, ^3J = 7.9 Hz, ArH), 7.73-7.85 (m, 1H, ArH), 7.62 (d, 2H, ^3J = 8.6 Hz, ArH), 7.50 (d, 2H, ^3J = 8.6 Hz, ArH), 7.25-7.35 (m, 1H, ArH), 4.68 (t, 2H, ^3J = 6.0 Hz, CH_2), 3.75 (t, 2H, ^3J = 6.4 Hz, CH_2), 2.32 (quin, 2H, ^3J = 6.1 Hz, CH_2).

^{13}C NMR (63 MHz, $CDCl_3$): δ (ppm) 160.9, 159.0, 150.9, 149.4, 137.0, 131.8, 130.6, 128.3, 124.4, 120.5, 119.1, 113.6, 67.1, 41.6, 32.5.

MS (EI): *m*/*z* 409.9 [40%] M+2, 407.9 [28%] M, 333.9 [16%], 331.9 [16%], 263.9 [30%], 218.9 [100%].

HRMS: *m*/*z* calculated: 407.9699, found: 407.9698.

4-(3-Azidopropoxy)-5-(4-bromophenyl)-2-(pyridine-2-yl)thiazole (BPT, 1)

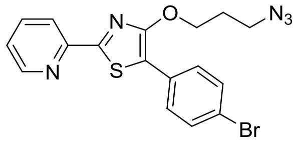

In a 100 mL round bottom flask 0.84 g (2.1 mmol) **5**, 0.27 g (4.1 mmol) NaN_3 and 20 mL dimethyl formamide were stirred for 4 h at 80 °C. The cooled mixture was poured into 100 mL of water and extracted with CH_2Cl_2 (3 x 50 mL). The combined extracts were washed with water (100 mL) and dried over $MgSO_4$. The solvent was removed in vacuum and the product, bright yellow crystals, was dried with a vacuum pump for several hours (83% yield).

^{1}H NMR (250 MHz, $CDCl_3$): δ (ppm) 8.59 (d, 1H, ^4J = 4.6 Hz, ArH), 8.10 (d, 1H, ^3J = 7.9 Hz, ArH), 7.73-7.85 (m, 1H, ArH), 7.63 (d, 2H, ^3J = 8.6 Hz, ArH), 7.50 (d, 2H, ^3J = 8.6 Hz, ArH), 7.27-7.36 (m, 1H, ArH), 4.62 (t, 2H, ^3J = 6.1 Hz, CH_2), 3.53 (t, 2H, ^3J = 6.7 Hz, CH_2), 2.14 (quin, 2H, ^3J = 6.3 Hz, CH_2).

^{13}C NMR (63 MHz, $CDCl_3$): δ (ppm) 160.9, 158.9, 150.9, 149.4, 137.0, 131.8, 130.6, 128.3, 124.4, 120.5, 119.1, 113.6, 67.3, 48.4, 29.0.

MS (EI): *m/z* 417.0 [28%] M+2, 415.0 [28%] M, 333.9 [50%], 331.9 [50%], 200.7 [100%].

HRMS *m/z* calculated: 415.0103, found: 415.0096.

LC/MS (ESI, positive mode) *m/z* 416.0 $[M+H]^+$.

N-(3-Bromopropyl)-7-nitrobenzo[c][1,2,5]oxadiazol-4-amine

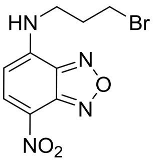

The substance was synthesized according to Key and Cairo [2].

^{1}H NMR (400 MHz, D_3COD): δ (ppm) 8.43 (d, 1H, ^3J = 8.8 Hz, ArH), 6.30 (d, 1H, ^3J = 8.8 Hz, ArH), 3.63 (m, 2H, CH_2-NH), 3.51 (t, 2H, ^3J = 6.0 Hz, CH_2-Br), 2.23 (quin, 2H, ^3J = 7.0 Hz, CH_2).

^{13}C NMR (100 MHz, D_3COD): δ (ppm) 146.6, 145.9, 145.5, 138.3, 123.5, 99.9, 43.2, 32.4, 31.0.

N-(3-Azidopropyl)-7-nitrobenzo[c][1,2,5]oxadiazol-4-amine (NBD, 9)

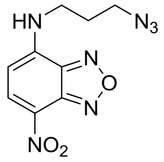

9 was synthesized according to Key and Cairo [2].

^{1}H NMR (400 MHz, $CDCl_3$): δ (ppm) 8.48 (d, 1H, ^{3}J = 8.4 Hz, ArH), 6.50 (m, 1H, NH), 6.21 (d, 1H, ^{3}J = 8.8 Hz, ArH), 3.63 (q, 2H, ^{3}J = 6.2 Hz, CH_2-NH), 3.58 (t, 2H, ^{3}J = 6.2 Hz, CH_2-N_3), 2.07 (quin, 2H, ^{3}J = 6.6 Hz, CH_2).

^{13}C NMR (100 MHz, $CDCl_3$): δ (ppm) 144.3, 143.8, 143.6, 136.4, 124.4, 98.7, 49.1, 41.56, 27.7.

LC/MS (ESI, positive mode) *m/z* 264.1 $[M+H]^+$.

N-(3-Azidopropyl)-5-(dimethylamino)naphthalene-1-sulfonamide (DNS, 8)

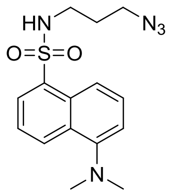

The synthesis of DNS was conducted with 5-(dimethylamino)naphthalene-1-sulfonyl chloride and 3-azidopropan-1-amine adapted from [3] who synthesized *N*-(2-azidoethyl)-5-(dimethylamino)naphthalene-1-sulfonamide. The crude product was purified by column chromatography using petroleum ether/ethyl acetate (2/1, v/v) and dried under reduced pressure resulting in 79% yield.

^{1}H NMR (400 MHz, $CDCl_3$): δ (ppm) 8.55 (d, 1H, ^{3}J = 8.5 Hz, ArH), 8.28 (d, 1H, ^{3}J = 8.6 Hz, ArH), 8.26 (dd, 1H, ^{3}J = 7.3 Hz, ^{4}J = 1.5 Hz, ArH), 7.51-7.59 (m, 2H,

ArH), 7.19 (d, 1H, ^3J = 7.3 Hz, ArH), 5.06 (m, 1H, NH), 3.25 (t, 2H, ^3J = 6.6 Hz, CH_2-N_3), 2.98 (q, 2H, ^3J = 6.2 Hz, N-CH_2), 2.89 (s, 6H, CH_3), 1.64 (quin, 2H, ^3J = 6.6 Hz, CH_2).

^{13}C NMR (101 MHz, $CDCl_3$): δ (ppm) 152.1, 134.3, 130.6, 129.9, 129.7, 129.5, 128.5, 123.2, 118.5, 115.2, 48.7, 45.4, 40.7, 28.7.

LC/MS (ESI, positive mode) *m*/*z* 334.2 $[M+H]^+$.

6-Bromo-5-(dimethylamino)naphthalene-1-sulfonyl chloride (7)

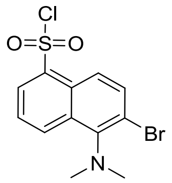

Starting form 5-dimethylamino-1-naphthalenesulfonyl chloride (dansyl chloride) the product was synthesized according to literature [4].

^{1}H NMR (600 MHz, $CDCl_3$): δ (ppm) 8.80 (d, 1H, ^3J = 8.3 Hz, ArH), 8.46 (d, 1H, ^3J = 8.8 Hz, ArH), 8.37 (dd, 1H, ^3J = 7.2 Hz, ^4J = 1.1 Hz, ArH), 7.87 (d, 1H, ^3J = 8.8 Hz, ArH), 7.65 (t, 1H, ^3J = 7.7 Hz, ArH), 3.04 (s, 6H, CH_3).

^{13}C NMR (151 MHz, $CDCl_3$): δ (ppm) 148.2, 139.9, 136.1, 134.9, 133.6, 129.5, 127.7, 124.6, 122.5, 122.1, 42.6.

LC/MS (ESI, positive mode) *m*/*z* 348.0 $[M+H]^+$.

***N*-(3-Azidopropyl)-6-bromo-5-(dimethylamino)naphthalene-1-sulfonamide (BNS, 6)**

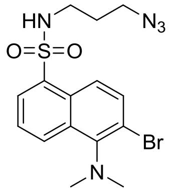

166 mg (0.48 mmol) 6-Bromo-5-(dimethylamino)naphthalene-1-sulfonyl chloride (**7**) were treated with 70 µl (0.71 mmol) 3-azidopropan-1-amine and 123 µL (0.89 mmol) triethylamine in 40 mL CH_2Cl_2. After stirring over night the solvent was removed under reduced pressure. The product was isolated by column chromatography with petrol ether/ethyl acetate 2/1, (v/v) and a yellow oil was obtained with 93% yield.

^{1}H NMR (400 MHz, $CDCl_3$): δ (ppm) 8.64 (d, 1H, ^3J = 8.5 Hz, ArH), 8.33 (d, 1H, ^3J = 8.8 Hz, ArH), 8.27 (dd, 1H, ^3J = 7.3 Hz, ^4J = 1.2 Hz, ArH), 7.74 (d, 1H, ^3J = 9.5 Hz, ArH), 7.60 (dd, 1H, ^3J = 8.8 Hz, ^3J = 7.2 Hz, ArH), 4.89 (t, 1H, ^3J = 6.3 Hz, NH), 3.30 (t, 2H, ^3J = 6.5 Hz, CH_2-N_3), 3.03 (s, 6H, CH_3), 3.01 (q, 2H, ^3J = 6.4 Hz, N-CH_2), 1.68 (quin, 2H, ^3J = 6.4 Hz, CH_2).

^{13}C NMR (100 MHz, $CDCl_3$): δ (ppm) 148.1, 135.9, 134.7, 133.8, 131.0, 130.0, 128.4, 124.9, 122.7, 121.3, 48.8, 42.6, 40.8, 28.8.

LC/MS (ESI, positive mode) *m*/*z* 412.1 $[M+H]^+$.

(2*E*,4*E*)-deca-2,4-dien-9-ynal (DDY, 10)

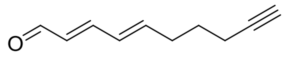

10 was synthesized as described elsewhere [5].

References

1. Täuscher, E.; Weiß, D.; Beckert, R.; Görls, H. *Synthesis* **2010,** 1603-1608.
2. Key, J. A.; Cairo, C. W. *Dyes Pigm.* **2011,** *88*, 95-102.
3. Rogers, S. A.; Bero, J. D.; Melander, C. *ChemBioChem* **2010,** *11*, 396–410.
4. Kinsey, B. M.; Kassis, A. I. *Nucl. Med. Biol.* **1993,** *20*, 13-22.
5. Wolfram, S.; Nejstgaard, J.; Pohnert, G *PLOSone*, in press.

Mass spectra of synthetic compounds

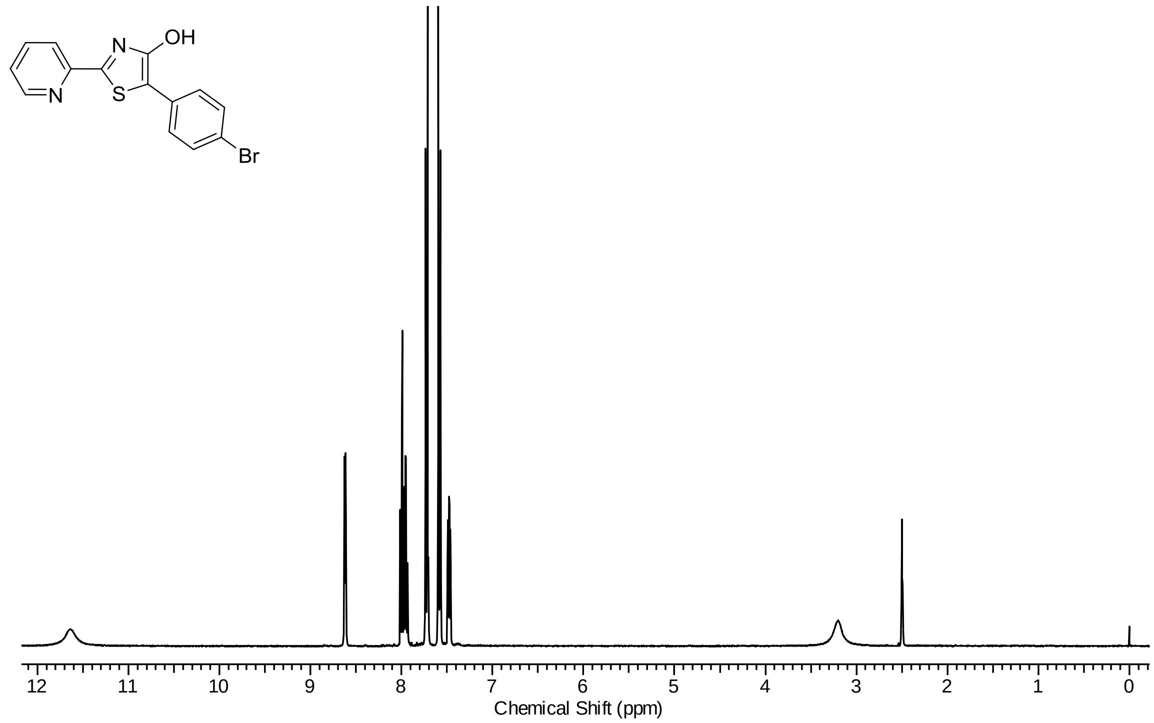

Figure 1: ^{1}H NMR spectrum of compound **4**.

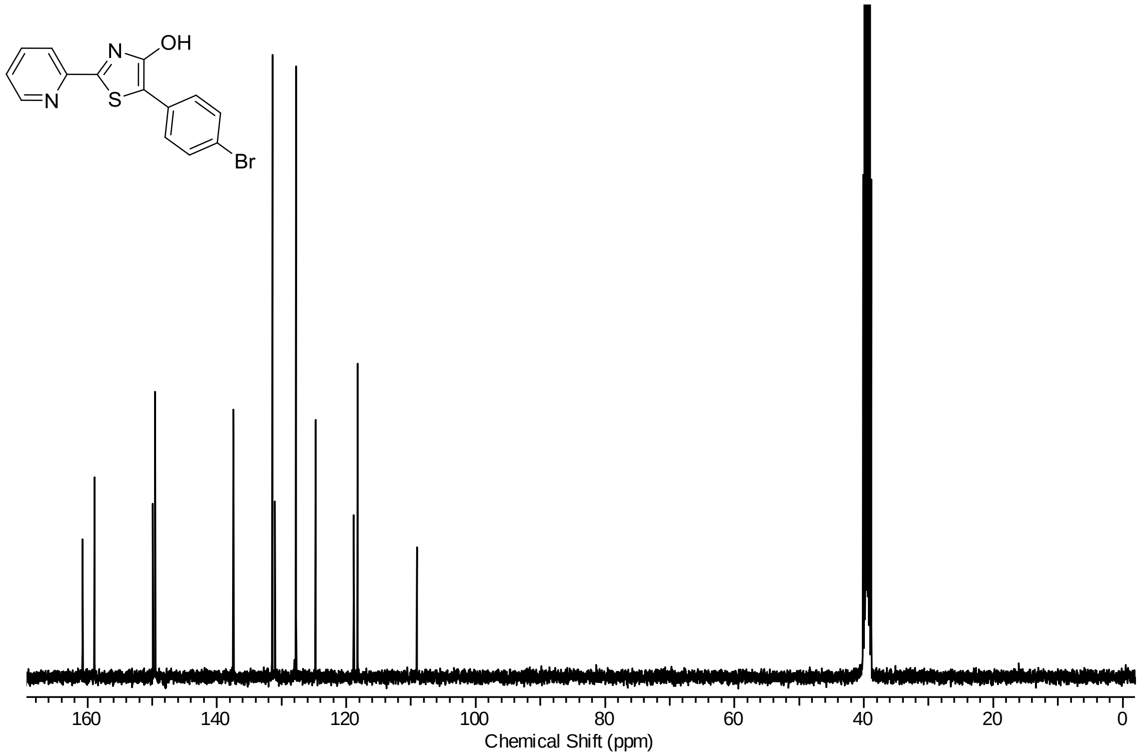

Figure 2: ^{13}C NMR spectrum of compound **4**.

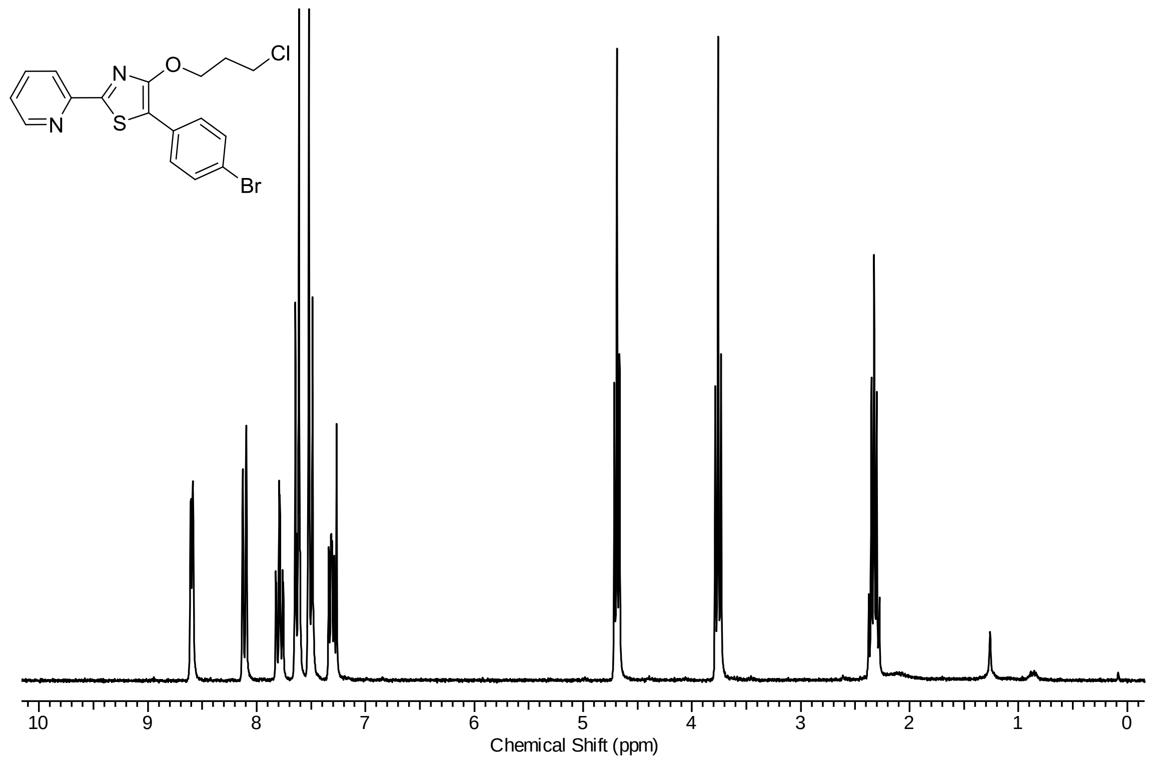

Figure 3: ^{1}H NMR spectrum of compound **5**.

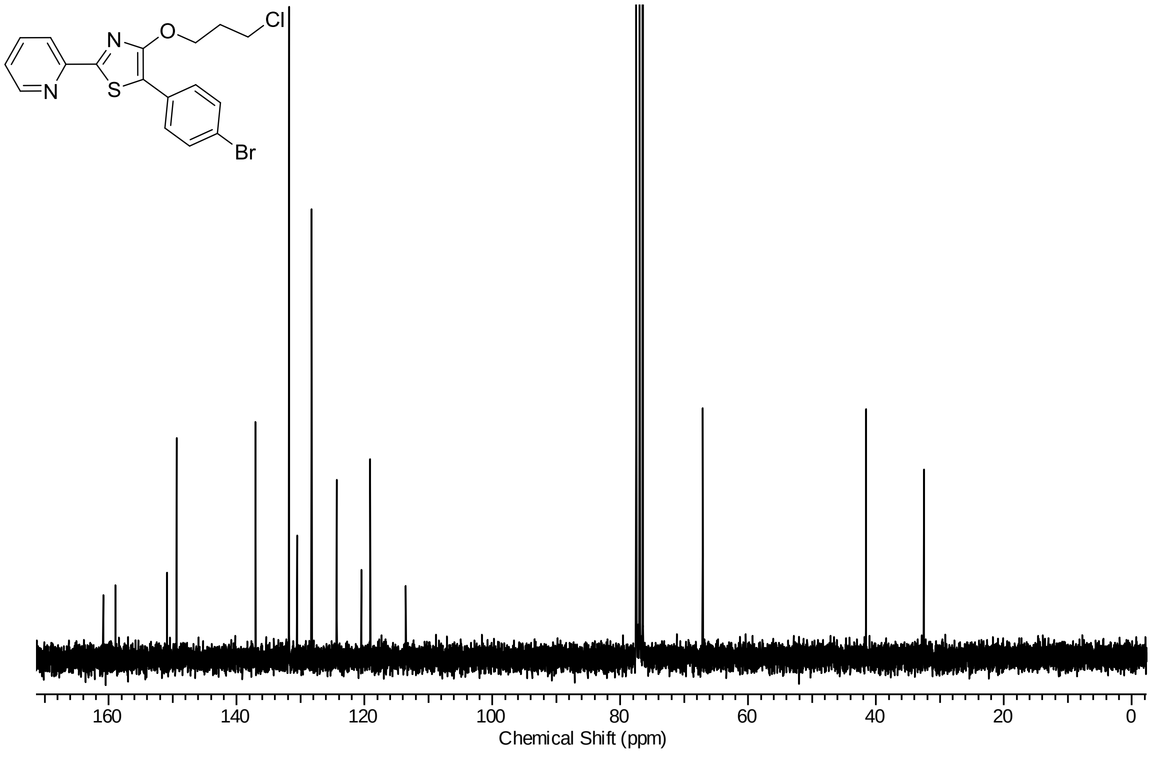

Figure 4: ^{13}C NMR spectrum of compound **5**.

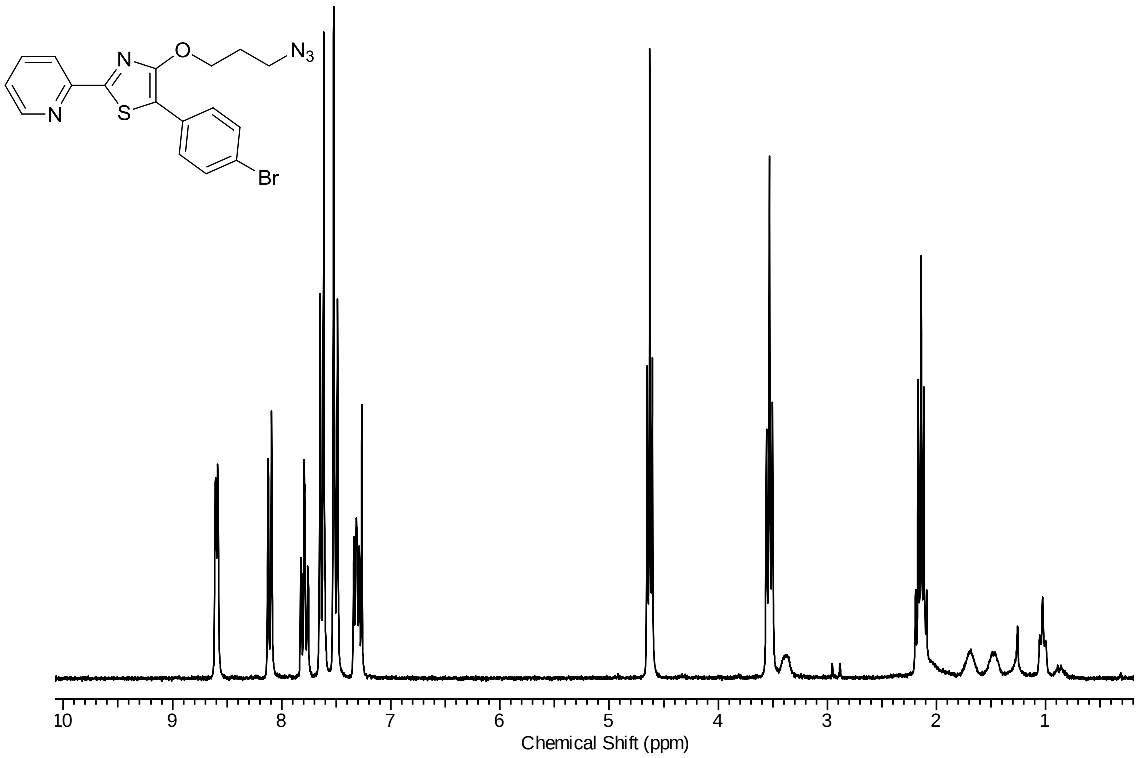

Figure 5: ^{1}H NMR spectrum of compound **1**.

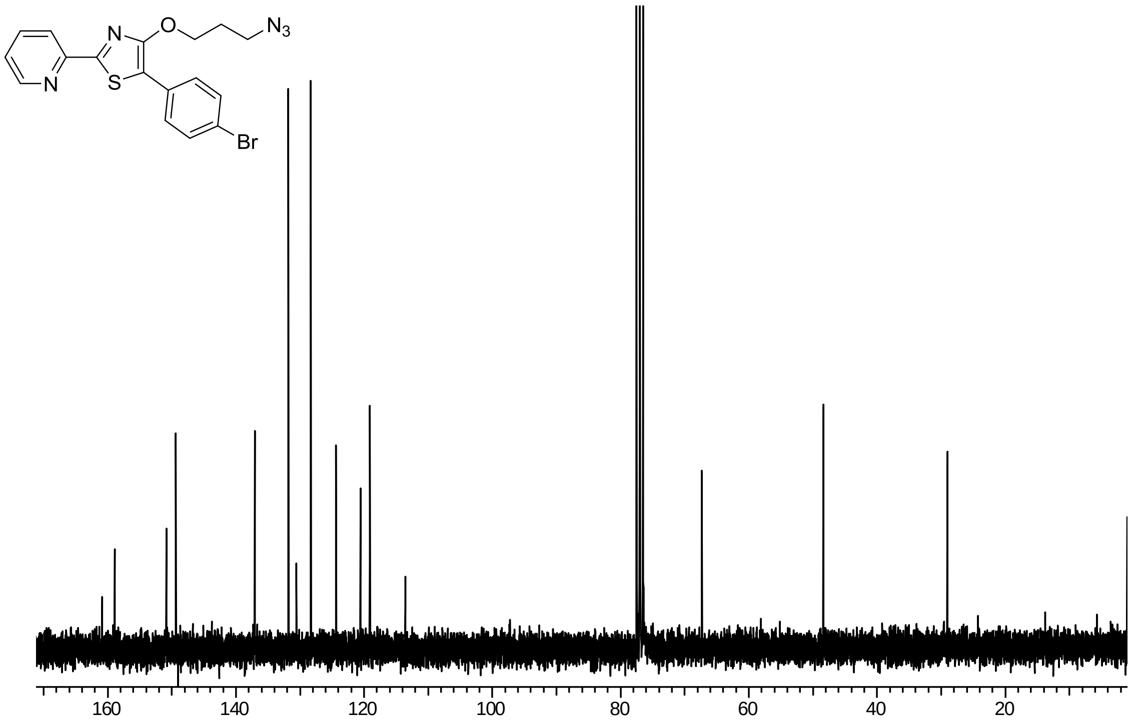

Figure 6: ^{13}C NMR spectrum of compound **1**.

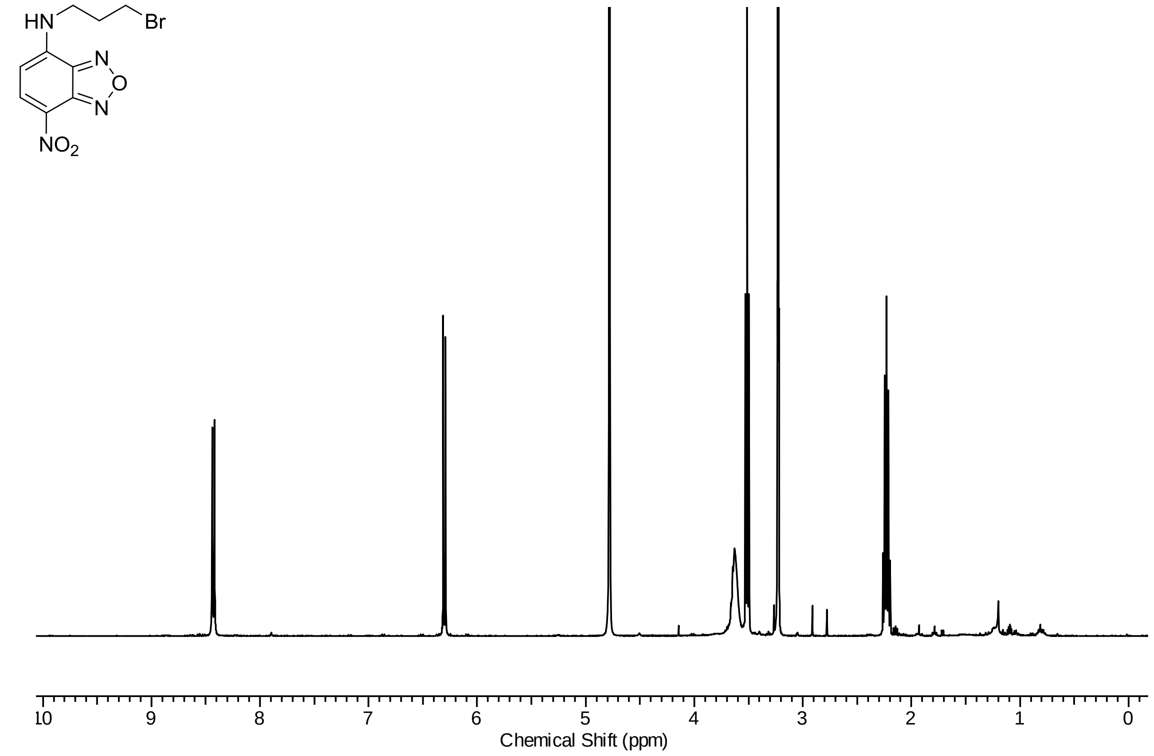

Figure 7: ^{1}H NMR spectrum of *N*-(3-bromopropyl)-7-nitrobenzo[*c*][1,2,5]oxadiazol-4-amine.

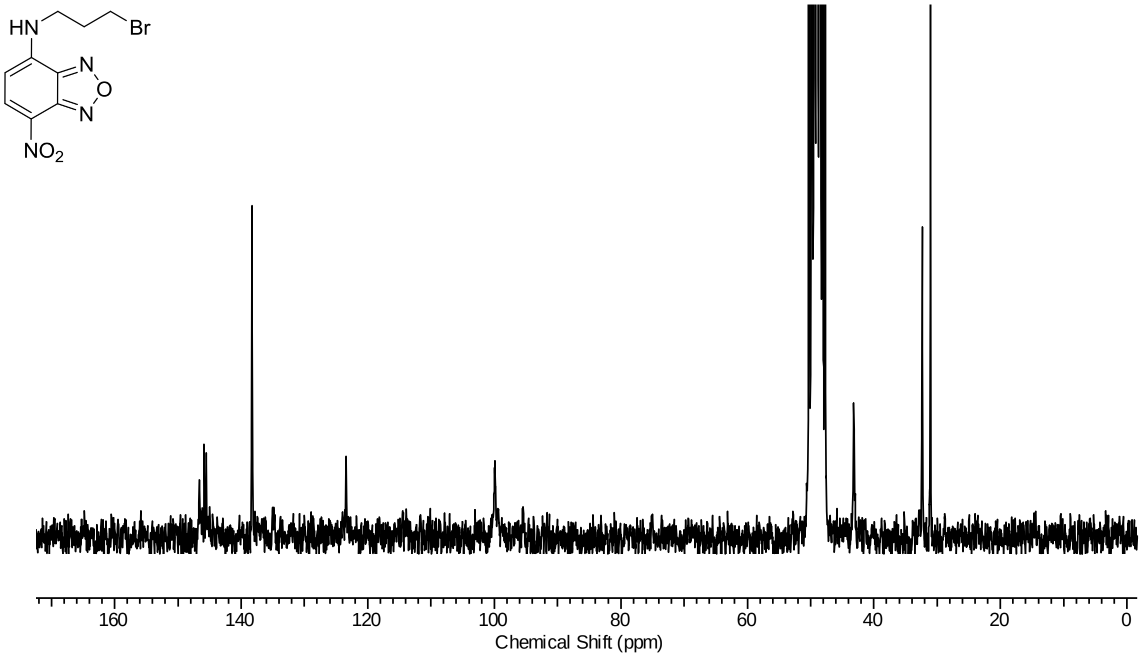

Figure 8: ^{13}C NMR spectrum of *N*-(3-bromopropyl)-7-nitrobenzo[*c*][1,2,5]oxadiazol-4-amine.

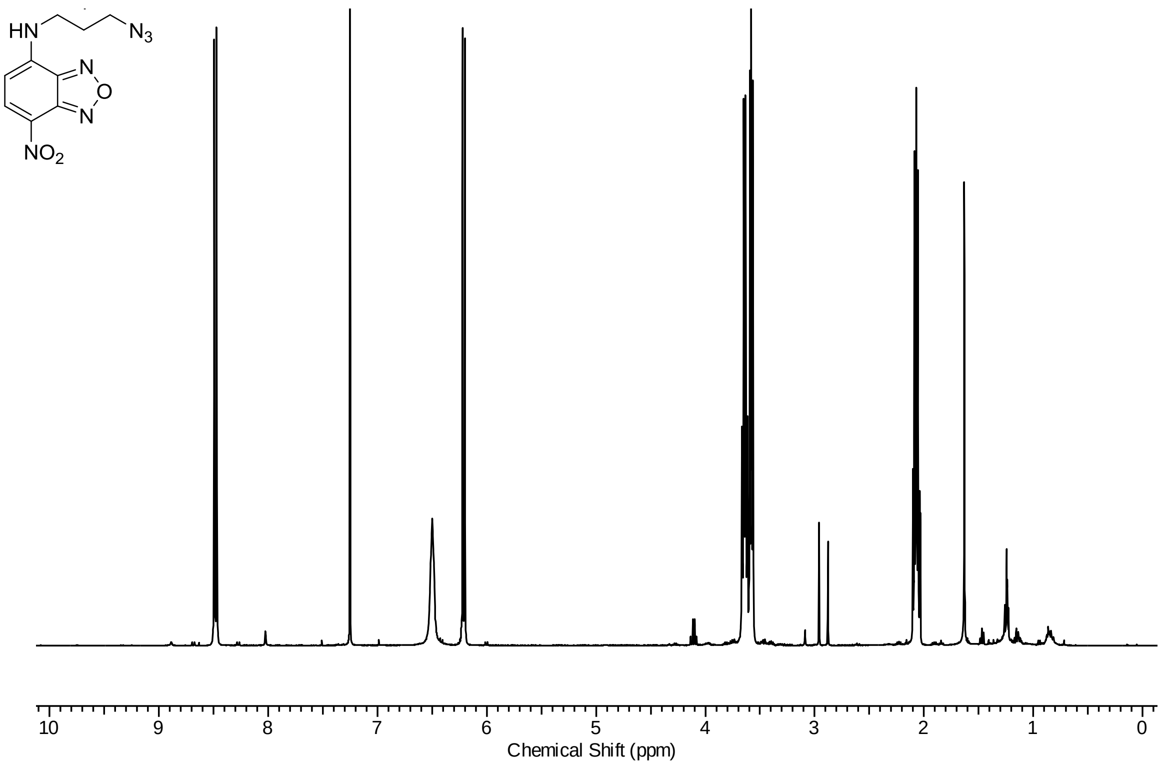

Figure 9: ^{1}H NMR spectrum of compound **9**.

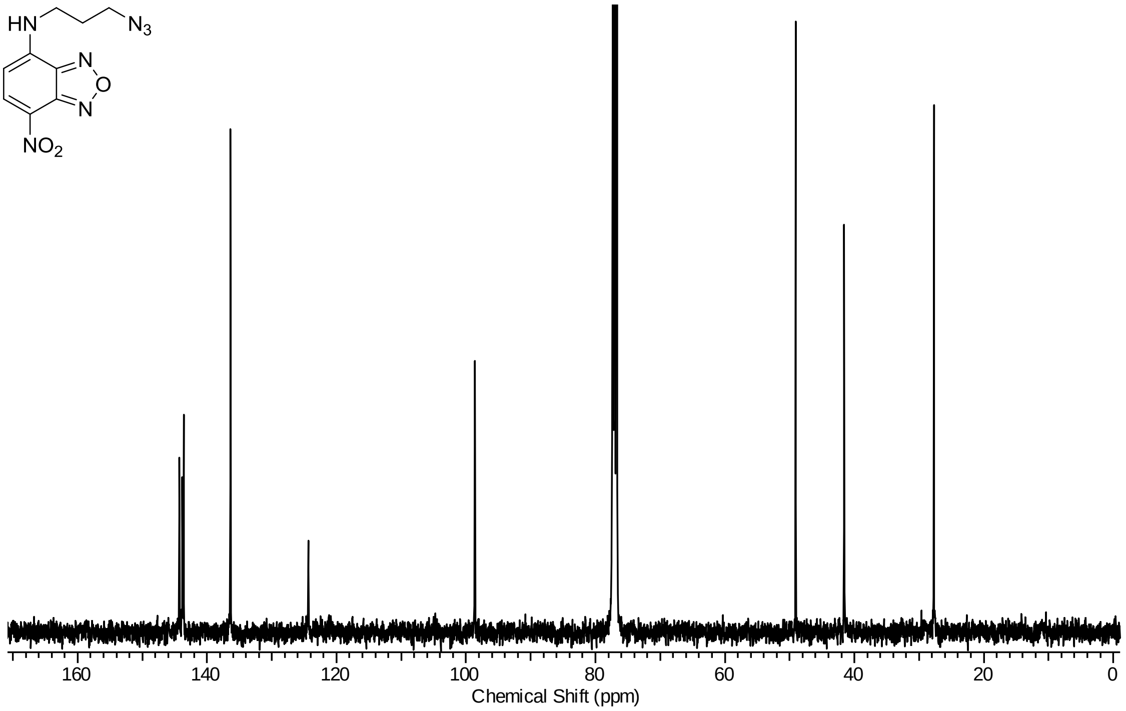

Figure 10: ^{13}C NMR spectrum of compound **9**.

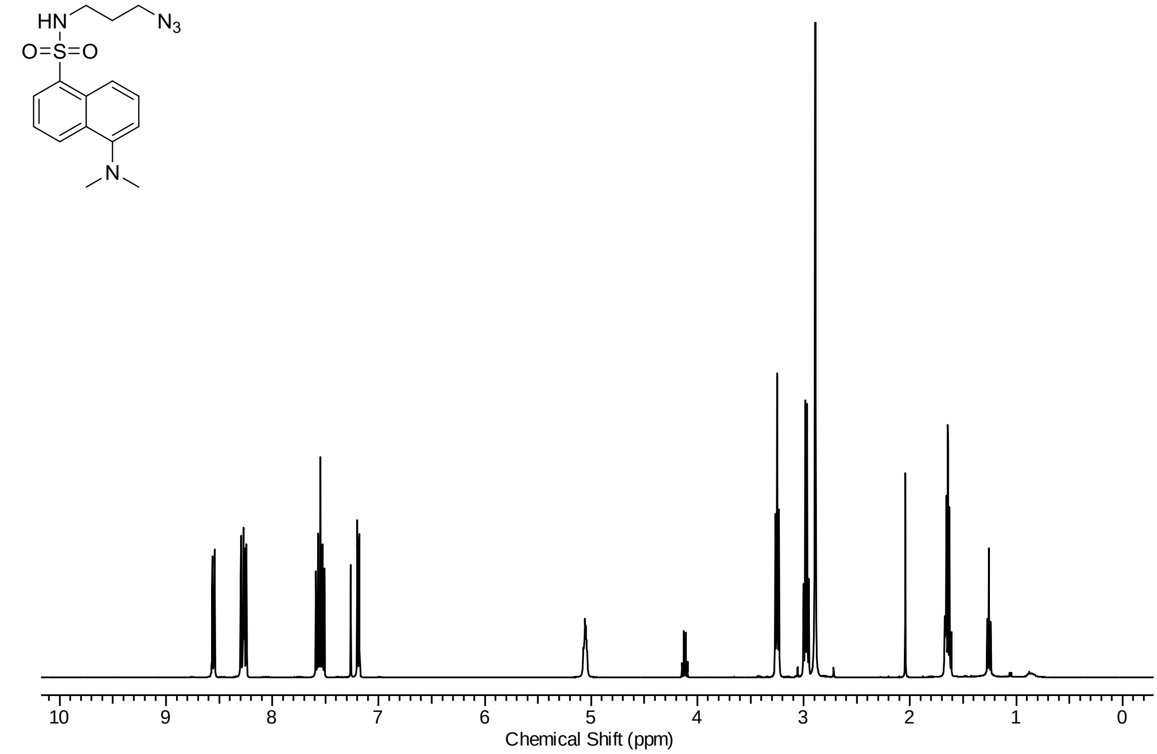

Figure 11: ^{1}H NMR spectrum of compound **8**.

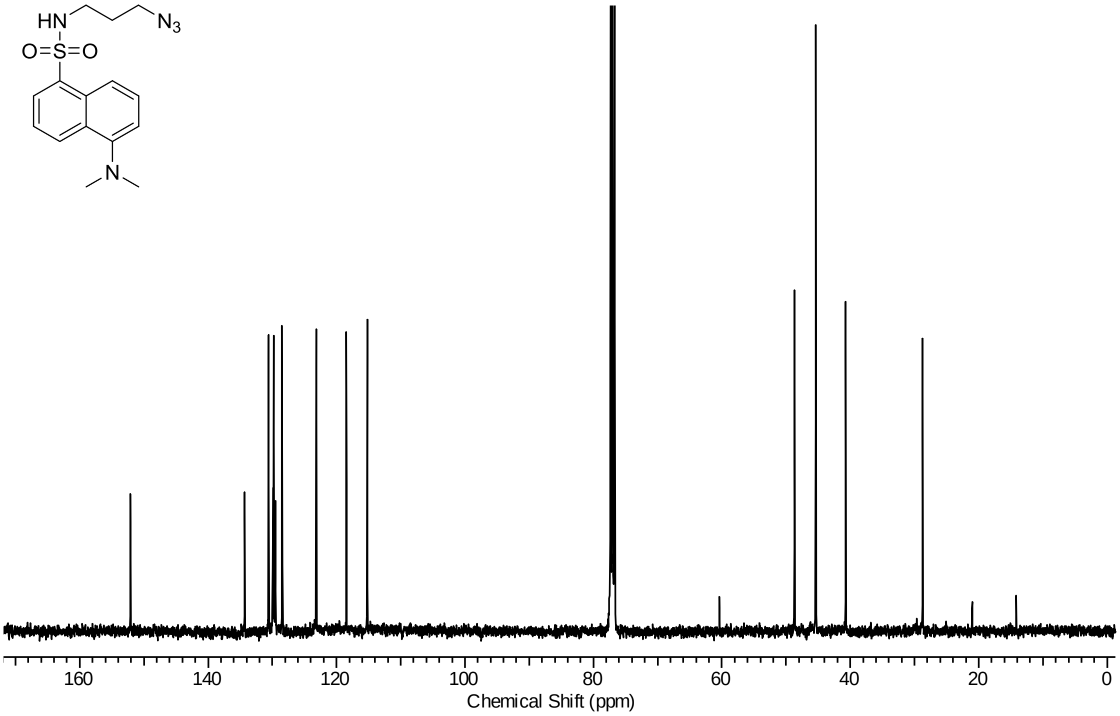

Figure 12: ^{13}C NMR spectrum of compound **8**.

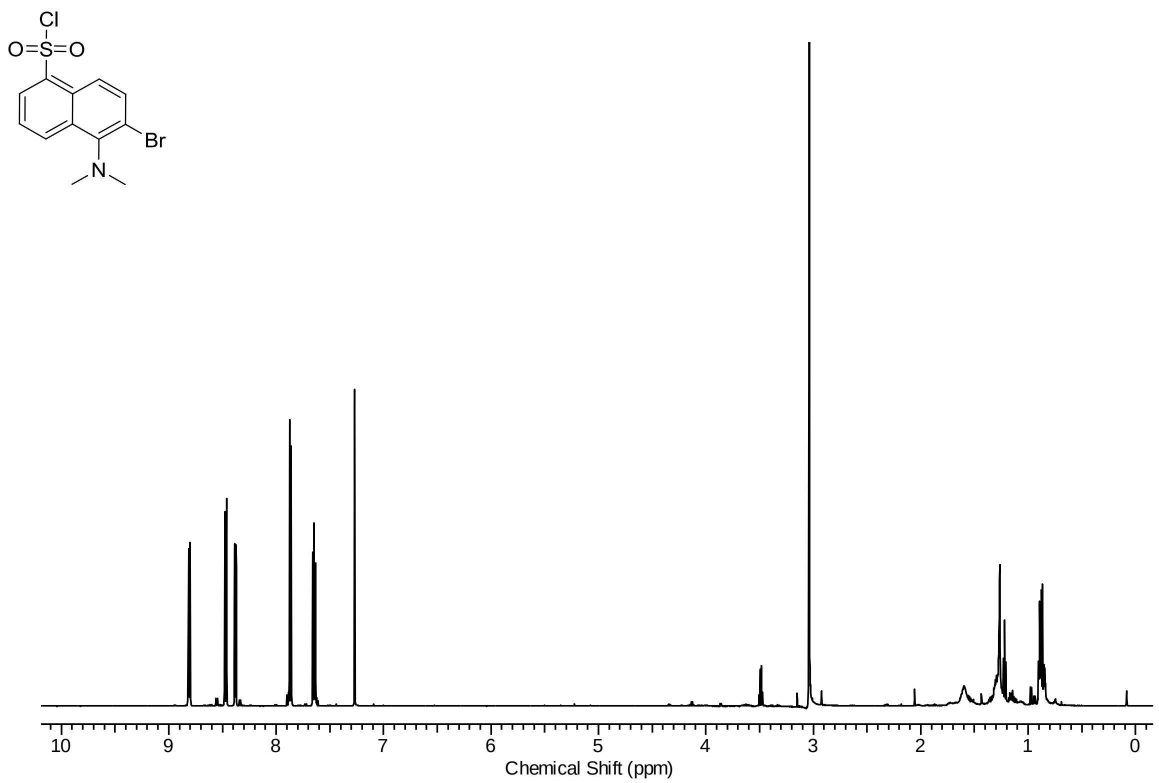

Figure 13: ^{1}H NMR spectrum of compound **7**.

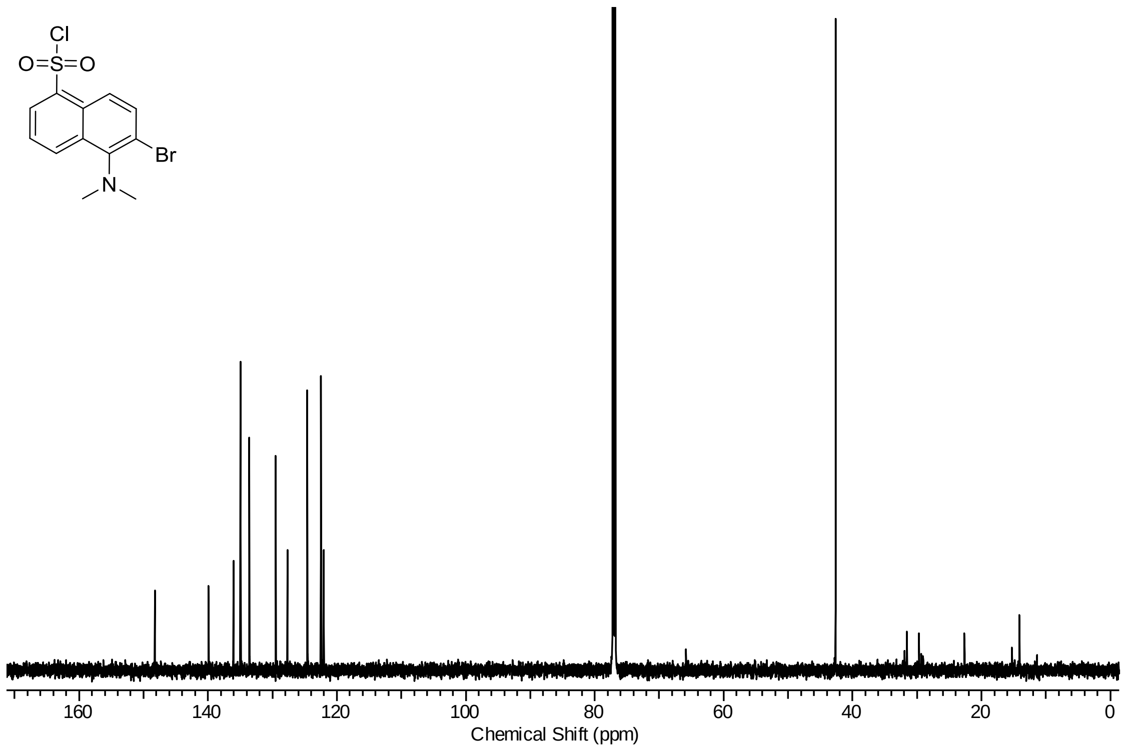

Figure 14: ^{13}C NMR spectrum of compound **7.**

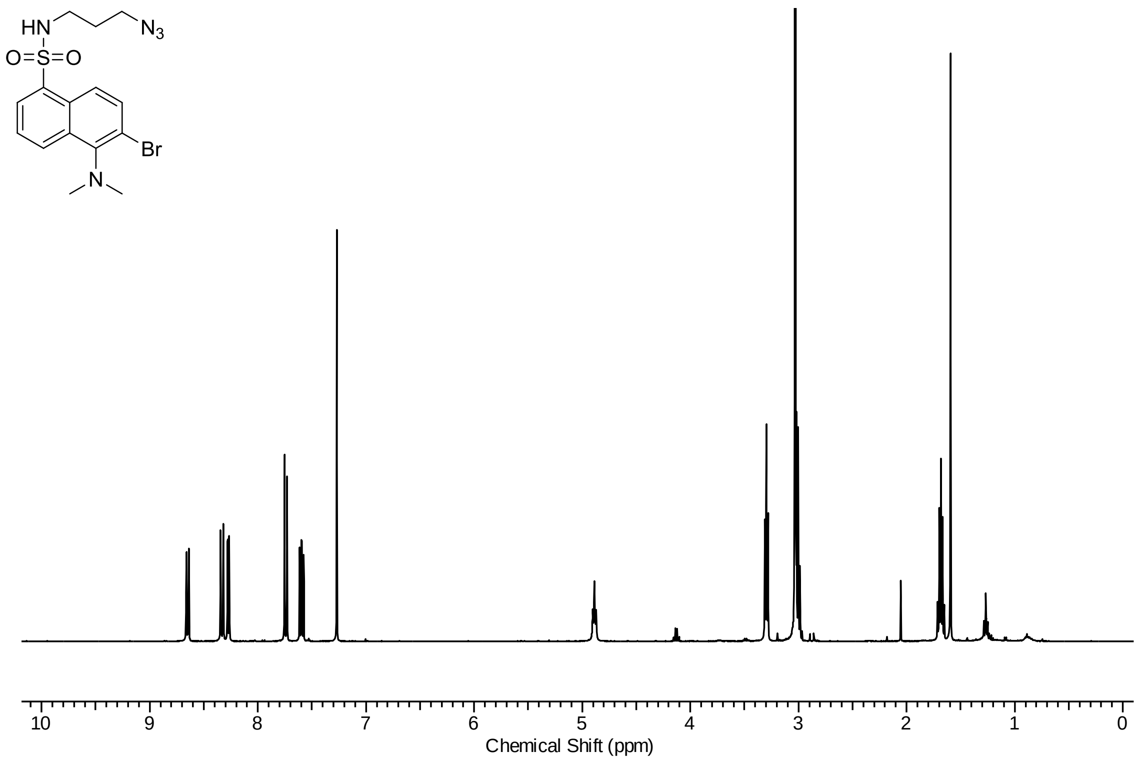

Figure 15: ^{1}H NMR spectrum of compound **6**.

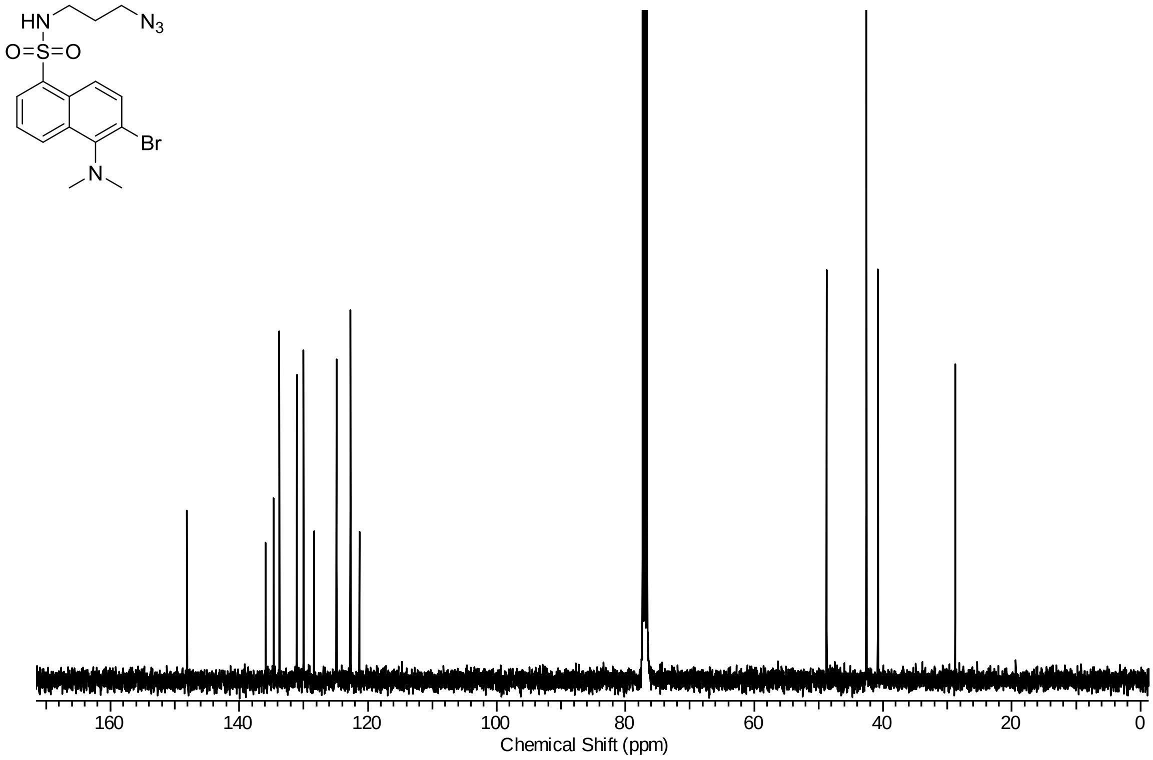

Figure 16: ^{13}C NMR spectrum of compound **6**.

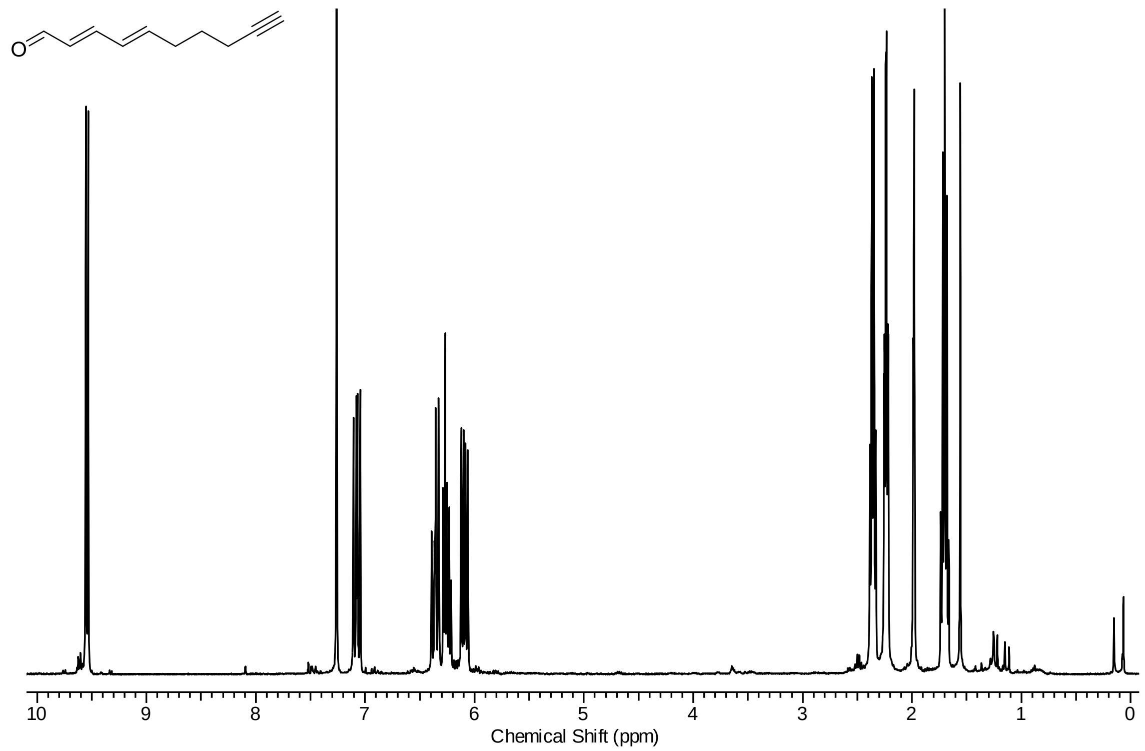

Figure 17: ^{1}H NMR spectrum of compound **10**.

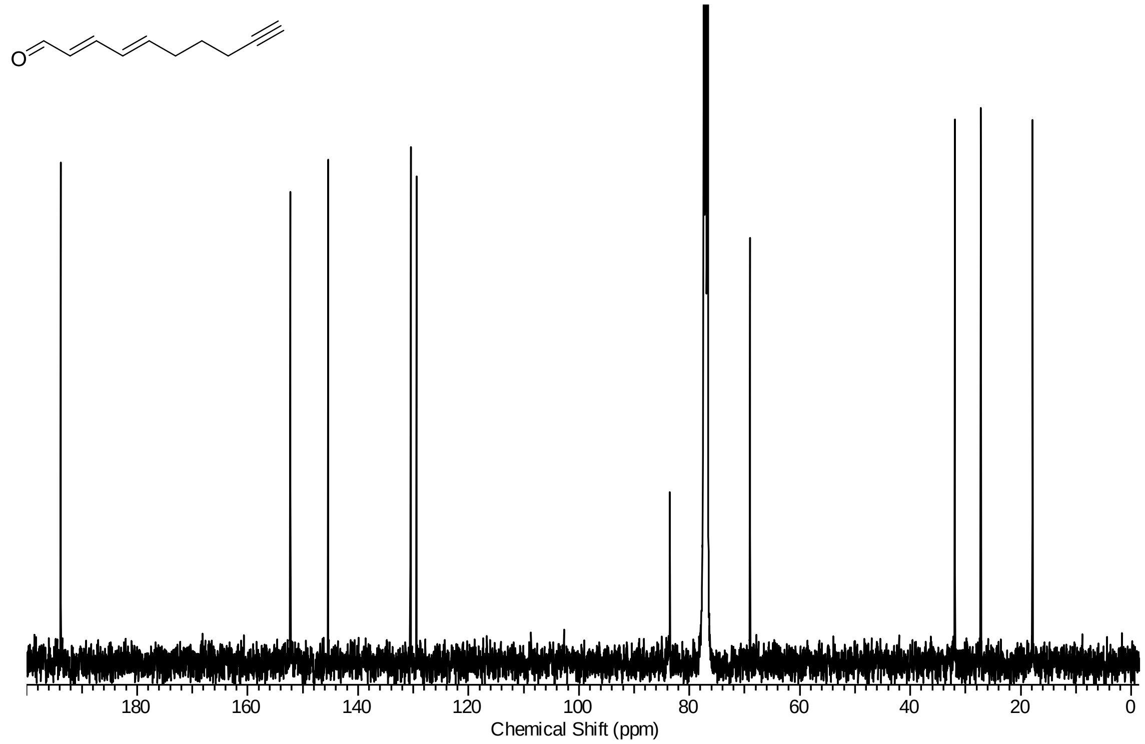

Figure 18: ^{13}C NMR spectrum of compound **10**.
